# Predictive biomarkers for 5‐ALA‐PDT can lead to personalized treatments and overcome tumor‐specific resistances

**DOI:** 10.1002/cnr2.1278

**Published:** 2020-07-31

**Authors:** Maria Mastrangelopoulou, Mantas Grigalavicius, Tine H. Raabe, Ellen Skarpen, Petras Juzenas, Qian Peng, Kristian Berg, Theodossis A. Theodossiou

**Affiliations:** ^1^ Department of Radiation Biology Institute for Cancer Research, Oslo University Hospital Oslo Norway; ^2^ Department of Molecular Cell Biology Institute for Cancer Research, Oslo University Hospital Oslo Norway; ^3^ Department of Pathology The Norwegian Radium Hospital, Oslo University Hospital Oslo Norway

**Keywords:** 5‐aminolevulinic acid, ABCG2 transporters, antioxidant defenses, ferrochelatase, heme oxygenase, metabolism, personalized therapy, photodynamic therapy

## Abstract

**Background:**

Photodynamic therapy (PDT) is a minimally invasive, clinically approved therapy with numerous advantages over other mainstream cancer therapies. 5‐aminolevulinic acid (5‐ALA)‐PDT is of particular interest, as it uses the photosensitiser PpIX, naturally produced in the heme pathway, following 5‐ALA administration. Even though 5‐ALA‐PDT shows high specificity to cancers, differences in treatment outcomes call for predictive biomarkers to better stratify patients and to also diversify 5‐ALA‐PDT based on each cancer's phenotypic and genotypic individualities.

**Aims:**

The present study seeks to highlight key biomarkers that may predict treatment outcome and simultaneously be exploited to overcome cancer‐specific resistances to 5‐ALA‐PDT.

**Methods and Results:**

We submitted two glioblastoma (T98G and U87) and three breast cancer (MCF7, MDA‐MB‐231, and T47D) cell lines to 5‐ALA‐PDT. Glioblastoma cells were the most resilient to 5‐ALA‐PDT, while intracellular production of 5‐ALA‐derived protoporphyrin IX (PpIX) could not account for the recorded PDT responses.

We identified the levels of expression of ABCG2 transporters, ferrochelatase (FECH), and heme oxygenase (HO‐1) as predictive biomarkers for 5‐ALA‐PDT. GPX4 and GSTP1 expression vs intracellular glutathione (GSH) levels also showed potential as PDT biomarkers.

For T98G cells, inhibition of ABCG2, FECH, HO‐1, and/or intracellular GSH depletion led to profound PDT enhancement. Inhibition of ABCG2 in U87 cells was the only synergistic adjuvant to 5‐ALA‐PDT, rendering the otherwise resistant cell line fully responsive to 5‐ALA‐PDT.

ABCG2 or FECH inhibition significantly enhanced 5‐ALA‐PDT‐induced MCF7 cytotoxicity, while for MDA‐MB‐231, ABCG2 inhibition and intracellular GSH depletion conferred profound synergies. FECH inhibition was the only synergism to ALA‐PDT for the most susceptible among the cell lines, T47D cells.

**Conclusion:**

This study demonstrates the heterogeneity in the cellular response to 5‐ALA‐PDT and identifies biomarkers that may be used to predict treatment outcome. The study also provides preliminary findings on the potential of inhibiting specific molecular targets to overcome inherent resistances to 5‐ALA‐PDT.

## 1 INTRODUCTION

Current anticancer treatment modalities, such as ionizing radiation, chemotherapeutics, hormone‐based therapies, and immunological treatments, risk failing as they encounter primary and secondary resistance, which varies not only from tumor to tumor but also within the same tumor, due to intrinsic heterogeneity.

Photodynamic therapy (PDT) is a minimally invasive, clinically approved therapy that uses three key components: a photoreactive drug (photosensitizer, PS), light of the appropriate wavelength, and oxygen. Upon light‐activation, the PS interacts with ambient molecular oxygen, generating highly reactive oxygen species (ROS) and prominently singlet oxygen (^1^O_2_), which facilitate cancer cell death.^1^, ^2^, ^3^, ^4^

PDT has numerous advantages over other mainstream cancer therapies, including cosmetic outcome, curative potential, and palliation to ensure improved quality of life, while it can be repeated as many times as required due to its minimal toxicity, low risk of treatment resistance, high treatment specificity, and low carcinogenic potential.^5^ Differences in treatment outcomes seen both preclinically and in patients call for predictive biomarkers to better stratify patients for PDT or alternative treatment modalities.

5‐aminolevulinic acid (5‐ALA)‐PDT is a particularly attractive form of PDT, since 5‐ALA is a natural intermediate in the heme synthesis that induces accumulation of the PS protoporphyrin IX (PpIX) upon administration as a prodrug to neoplastic cells. Iron (Fe^2+^) is incorporated into PpIX to form heme by means of the enzyme ferrochelatase (FECH). Heme, unlike PpIX, lacks PS properties, since the chelation of iron mutes both fluorescence and singlet oxygen generation. The main asset of 5‐ALA‐PDT lies in its specificity for cancer cells, as PpIX formation is much higher in tumor cells.^5^ This has been mainly attributed to the significantly lower FECH, located in the inner mitochondrial membrane,^6^ and enhanced porphobilinogen deaminase (PBGD) activity in cancer cells, leading to reduced conversion of PpIX into heme.^7^, ^8^, ^9^, ^10^, ^11^ However, other factors, such as limited access to free iron, may also contribute to the high specificity for PpIX accumulation in cancer cells.^12^ PpIX is converted to heme within 24 hours after 5‐ALA administration, causing no long‐term skin photosensitivity to the patients, one of the side‐effects associated with PDT. Moreover, 5‐ALA, being a hydrophilic and relatively small molecule (<200 Da), is in principle much easier to administer systemically than other larger and lipophilic PSs frequently used for clinical PDT.

Due to these attractive features, 5‐ALA‐PDT was first introduced to the clinic in 1990 (Kennedy et al^13^) and has since become an established, yet underrated, clinical cancer treatment option primarily for dermatological malignancies. In addition, 5‐ALA‐derived PpIX photodynamic diagnosis has already been clinically applied for the diagnosis of bladder,^14^ prostate,^15^ and brain malignancies.^16^, ^17^ 5‐ALA was approved by the U.S. Food and Drug Administration (FDA) in 2017, as an adjunct for the visualization of malignant tissue, in grade III or IV glioma (NDA 208630/SN0014).

Glioblastoma multiforme (GBM), is an orphan, incurable brain cancer with poor patient prognosis and consequent high mortality. The main reasons behind the unfavorable GBM progression are: (a) extensive infiltration of the tumor cells into the surrounding brain tissue limiting the efficiency of surgical resection and (b) innate or rapidly acquired tumor resistance to chemotherapy and radiotherapy.^18^, ^19^ At present, the standard of care for GBM comprises surgery, radiotherapy, and chemotherapy, giving a limited survival benefit to the patient however (approximately 15 months median survival vs 3 months when untreated).^20^ Early clinical studies indicate that 5‐ALA‐PDT is safe and potentially beneficial in treating GBM.^21^, ^22^

Breast cancer, by contrast, is the most common cancer in women, accounting for 30% of all female cancers, and an eminent cause of female morbidity and mortality.^23^ Standard treatment regimens for breast cancer include surgery, chemotherapy, immunotherapy, and radiation therapy, which have offered significant advances in primary disease treatment; however, refractory disease and recurrence remain prevalent.^24^ Depending on their expression of therapy‐related biomarkers, breast cancers can be classified as estrogen‐ and/or progesterone‐receptor positive, human epidermal growth factor‐receptor 2‐positive, and triple‐negative breast cancer (TNBC), which lacks the aforementioned receptors/markers.^25^ TNBC is the most difficult to cure, displaying a higher risk for drug resistance and cancer recurrence.^24^

We present an in vitro investigation of the outcome of 5‐ALA‐PDT on two human GBM cell lines, T98G and U87, and three breast adenocarcinoma cell lines, MCF7, MDA‐MB‐231, and T47D, and correlate the outcomes with the inherent characteristics of these cell lines. More specifically, we look into how 5‐ALA‐PDT‐induced cell death is affected by critical factors modulating the production of PpIX and its conversion into heme, cell metabolism, inherent antioxidant cell defenses, and mechanisms facilitating or inhibiting the expulsion of PpIX from the cells. The aim of the study is to establish crucial biomarkers, specific for 5‐ALA‐PDT, which will ultimately provide patient‐specific prognosis for treatment outcomes and which may lead to personalized treatments based on 5‐ALA‐PDT with profoundly higher efficacy, even in cells initially non‐responsive to 5‐ALA‐PDT alone.

## 2 METHODS

### 2.1 Chemicals and reagents

5‐ALA, dimethyl sulfoxide (DMSO), oligomycin (oligo), carbonyl cyanide 4‐(trifluoromethoxy)phenylhydrazone (FCCP), myxothiazol (MYXO), sodium pyruvate, β‐Nicotinamide adenine dinucleotide reduced (NADH), buthionine sulfoximine (BSO), Triton X‐100, polyethylene glycol sorbitan monolaurate (TWEEN 20), thiazolyl blue tetrazolium bromide (MTT), metaphosphoric acid (MPA), triethanolamine (TEAM), anti‐γ‐tubulin, Ko143 hydrate, protease inhibitor cocktail, phosphatase inhibitor cocktail I and II, RPMI 1640 without phenol red, l‐glutamine, penicillin‐streptomycin, trypsin and Dulbecco's phosphate‐buffered saline (PBS) with or without calcium chloride and magnesium chloride were purchased from Sigma‐Aldrich Norway AS (Oslo, Norway). Noc‐18, anti‐FECH, and anti‐superoxide dismutase 2 (SOD2) were purchased from Santa Cruz Biotechnology, Inc., (Dallas, Texas), and OB24 hydrochloride from Bio‐Techne Ltd (Abingdon, UK). Fetal bovine serum (FBS), Pierce lane marker reducing sample buffer, TrypLE express enzyme, and MitoTracker red CMXRos were purchased from Thermo Fisher Scientific (Oslo, Norway). Precision Plus Protein dual color standard and 12% Mini‐PROTEAN TGX precast proteins gels were purchased from Bio‐Rad (Hercules, California). Immobilon Western Chemiluminescent HRP Substrate was purchased from Merck (Oslo, Norway). Anti‐HO‐1, anti‐GPX4, and anti‐BCRP/ABCG2 antibodies were purchased from Abcam (Cambridge, UK). Anti‐superoxide dismutase 1 (SOD1) and anti‐glutathione‐S‐transferase 1 (GSTP1) were purchased from Cell Signaling Technology, Inc. (Danvers, Massachusetts). The total glutathione (GSH) assay kit was purchased from Cayman Chemical Company (Ann Arbor, Michigan). The consumables used for the Seahorse XFe96 metabolic analysis were purchased from Agilent Technologies (Santa Clara, California).

### 2.2 Cell culture

The cells used in this study were the human GBM cell lines T98G (ATCC CRL‐1690) and U87 (ATCC HTB‐14) and the human breast cancer cell lines MCF7 (ATCC HTB‐22), MDA‐MB‐231 (ATCC HTB‐26), and T47D (ATCC HTB‐133). Since all cell lines were purchased from ATCC (LGC Standards GmbH, Wesel, Germany), no additional authentication was carried out; all cells were tested negative for mycoplasma (MycoAlert, Lonza Group Ltd, Basel, Switzerland). The cells were grown in RPMI 1640 without phenol red, supplemented with 10% FBS, 100 U/mL penicillin/100 μg/mL streptomycin and 2 mm l‐Glutamine at 37°C in a 5% CO_2_ humidified atmosphere.

### 2.3 PDT experiments and light irradiation

The cells were seeded in 96‐well plates (Nunc, 1.5 × 10^4^ cells/100 μL media/well or Agilent Seahorse, 3 × 10^4^ cells/100 μL media/well) and incubated overnight in complete media. The cells were incubated with various concentrations of 5‐ALA (0.1‐2 mM) in Opti‐MEM (Thermo Fisher Scientific) for 4 hours. Prior to irradiation, the cells were washed twice with PBS containing calcium and magnesium, and complete media were added to the cells. The cells were irradiated from the underside of the plate with blue light by means of an in‐house‐built lamp that was equipped with LEDs at 405 nm. The irradiance at the cell level was 5.6 mW/cm^2^, as measured by an irradiance‐calibrated AvaSpec‐2048x14‐SPU2 FiberOptic Spectrometer (Avantes, Apeldoorn, The Netherlands). Varying irradiation times were used (0‐120 seconds), resulting in different light doses. In all the experiments, a light positive control without 5‐ALA and dark controls with and without 5‐ALA were included.

### 2.4 Cell treatment with various inhibitors prior to 5‐ALA‐PDT

**BSO treatment:** BSO (100 μM) was added to the cells overnight prior to incubation with 5‐ALA and maintained in the respective treatment groups until the assessment of cytotoxicity (20 hours after irradiation). The cells were incubated with 5‐ALA (0.2 mM T47D and MDA‐MB‐231, 0.5 mM MCF7, 1 mM T98G,and 2 mM U87) for 4 hours, washed twice in PBS, and irradiated at different time points per cell line to achieve LD_30_ for each cell line (120 seconds T47D, 90 seconds MDA‐MB‐231, 60 seconds MCF7, 60 seconds U87, and 120 seconds T98G).

**Treatment with other inhibitors:** Ko143 (1 or 5 μM) was added to the cells 1 hour prior to incubation with 5‐ALA and maintained in the respective groups until the assessment of cytotoxicity (20 hours after irradiation). Noc‐18 (0.5 mM) or OB24 (20 μM) were added to the cells either overnight or 1 hour prior to 5‐ALA addition and removed immediately prior to irradiation. The cell lines were incubated with different 5‐ALA concentrations and irradiated at different times to achieve LD_30_ conditions (see BSO treatment). A synergistic cytotoxicity of 5‐ALA‐PDT with the inhibitors was determined when *Viability (5‐ALA + inhibitor) < Viability (5‐ALA) × Viability (inhibitor)/ Viability (CTRL)*. In the cases where *Viability (5‐ALA + inhibitor) ~ Viability (5‐ALA) × Viability (inhibitor)/Viability (CTRL)*, the effects were merely additive.

### 2.5 Flow cytometry on 5‐ALA‐derived PpIX

The cells were seeded in 6‐well plates (Nunc, 7 × 10^5^ cells per well) and incubated overnight. Ko143 (1 μM) was added to the cells 1 hour prior to 5‐ALA addition and maintained in the respective groups until the end of the treatment (20 hours after irradiation). The cells were incubated with 5‐ALA (0.2 and 0.5 mM T47D and MDA‐MB‐231, 0.5 mM MCF7, 1 and 0.5 mM T98G, and 2 and 0.5 mM U87) for 4 hours, washed in PBS with calcium and magnesium, and trypsinated with 1 mL TrypLE Express. Next, the cells were resuspended in ice‐cold PBS containing calcium and magnesium and measured using an LSRII (BD) flow cytometer with excitation at 405 nm. The fluorescence was registered at the BV650 channel (635 nm cut‐on longpass filter and 660/20 nm bandpass filter). All flow cytometry data were analyzed in FlowJo v.10.6.2 (Treestar Inc., Ashland, Oregon).

### 2.6 Cell viability assessment

Twenty hours following irradiation, the redox function of all the cell groups was assessed by MTT assay. Briefly, 100 μL of complete media containing 0.5 mg/mL MTT was added to the cells and incubated for 3 hours at 37°C in a 5% CO_2_ incubator. Subsequently, the MTT media were removed, and 100 μL DMSO was added per well. The plates were shaken for 10 minutes at 350 rpm on a HeidolphTitramax 101 orbital shaker (Heidolph Instruments GmbH & Co. KG, Schwabach, Germany), and an endpoint absorbance measurement was performed at 561 nm using the Tecan spark M10 plate reader (Tecan Group Ltd., Männedorf, Switzerland). Blank values measured in wells with DMSO and no cells were subtracted in all cases.

### 2.7 Metabolic assay

T98G, U87, MCF7, MDA‐MB‐231, and T47D cells were seeded in Seahorse XFe96 96‐well plates (3 × 10^4^ cells per well) and incubated overnight in a 37°C, 5% CO_2_ humidified atmosphere. The next day, cells were incubated with various concentrations of 5‐ALA (0.5 mM T47D and MDA‐MB‐231, 1 mM MCF7, and 2 mM T98G and U87) for 4 hours and irradiated for 60 seconds for each cell line. One hour prior to metabolic measurement, the cells were placed in Seahorse XF RPMI media, pH 7.4 supplemented with 10 mM glucose, 2 mM sodium pyruvate, and 2 mM glutamine and incubated at 37°C in the absence of CO_2_. Cell respiration was monitored through the oxygen consumption rate (OCR) with a Seahorse XFe96 analyzer (Agilent, Santa Clara, California), before (basal rate) and after the sequential addition of respiration modulators (1 μM oligo, 1 μM FCCP, and 2 μM MYXO). The cells were also studied for their glycolytic activity by measuring the rate of extracellular acidification before (basal rate) and after the addition of 1 μM oligo.

### 2.8 Glutathione assay

The total GSH was measured using a glutathione assay kit (Cayman Chemical), following the Tietze recycling assay method^26^ and according to the manufacturer's instructions. Briefly, the cells were seeded in 96‐well plates (Nunc, 40 × 10^3^ cells/100 μL media/well) and incubated overnight in their normal media in a 37°C, 5% CO_2_ humidified atmosphere. BSO (100 μM) was added to cells overnight where appropriate. The cells were incubated with 5‐ALA (0.2 mM T47D and MDA‐MB‐231, 0.5 mM MCF7, 1 mM T98G, and 2 mM U87) for 4 hours in the presence and absence of BSO, washed twice in PBS with calcium and magnesium, and finally left in complete medium. One hour following the addition of the medium, the cells were either treated with 0.5% MPA (for the glutathione assay) or 0.1% (v/v) Triton‐X 100 aqueous solution for protein quantification. The cells were then kept at −20°C until the GSH assay was performed. Prior to the glutathione assay, 2 μL of 4 M TEAM was added to each well treated with MPA to reinstate the physiological pH (7.4). Part of the supernatant (50 μL) from each well was moved to a new 96‐well plate and assayed for GSH content. The kinetic absorbance measurement was performed at 405 nm using the Tecan spark M10 plate reader. Blank values measured in wells with no cells were subtracted in all cases.

### 2.9 Live cell imaging

The cells were seeded on glass bottom 35‐mm dishes (MatTek Corp., Ashland, Massachusetts) containing 2 × 10^5^ cells per dish and incubated overnight. The next day, Ko143 (1 μM) was added to cells 1 hour prior to 5‐ALA addition (1.5 mM ALA, 1 hour or 4 hours) and maintained in the respective groups until imaging. The mitochondrial probe MitoTracker Green FM (100 nm) was added to the cells 15 minutes prior to live cell imaging. The cells were examined with a Zeiss LSM 880 Airyscan microscope (Carl Zeiss MicroImaging GmbH, Jena, Germany), equipped with an Ar Laser Multiline (458/488/514 nm), a DPSS 561 10 (561 nm), a Laser diode 405‐30 CW (405 nm), and a HeNe Laser (633 nm). The objective used was a Zeiss plan‐Apochromat 63xNA/1.4 oil DICII. Image acquisition, processing and analysis were performed with ZEN 2.3 SP1 basic software (Carl Zeiss), ZEN 2.3 Blue (Carl Zeiss), or Imaris 7.7.2 (Bitplane AG, Zürich, Switzerland). The Pearson's colocalization measurements were performed in ZEN 2.3 Blue with Costes' automatic thresholding.

### 2.10 Western blotting

The cells were seeded on 60 mm dishes (Corning, 5 × 10^5^ cells per dish) and incubated overnight in complete media in a 37°C, 5% CO_2_ humidified atmosphere. The following day, the cells were incubated with 5‐ALA (0.2 mM T47D and MDA‐MB‐231, 0.5 mM MCF7, 1 mM T98G, and 2 mM U87) for 4 hours, washed twice with PBS with calcium and magnesium, and complete medium was reintroduced. The cells were irradiated (through the petri dish undersides) for different times to achieve LD_30_ for each cell line (120 seconds T47D, 90 seconds MDA, 60 seconds MCF7, 60 seconds U87, and 120 seconds T98G). Two hours following irradiation, the cells were lysed with RIPA buffer (also containing 10 μL/mL protease inhibitor cocktail, 10 μL/mL phosphatase inhibitor cocktail I and II, 10 μL/mL 2 M β‐glycerol phosphate, and 5 μL/mL NaF). Finally, lane marker sample buffer was added. The lysates were heated (95°C for 5 minutes), then applied on 18‐well 12% TGX gels together with the precision plus protein dual color standard, and subjected to sodium dodecyl sulfate‐polyacrylamide gel electrophoresis (120 V, 90 minutes). Subsequently, the proteins were blotted using the Trans‐Blot Turbo system onto PVDF transfer membranes (Bio‐Rad, Hercules, California). The membranes were washed with tris‐buffered saline containing 0.1% Tween 20 (TBST) and blocked in 5% milk for 1 hour at room temperature. The membranes were subsequently incubated with the primary antibodies in 5% milk overnight at 4°C. The following day, the membranes were washed in TBST and incubated with secondary antibodies in TBST for 1.5 hours at room temperature. The membranes were then washed with TBST and incubated with Immobilon Western Chemiluminescent HRP Substrate. Luminescence was measured with a ChemiDoc MP Imaging system (Bio‐Rad), and γ‐tubulin was used as the housekeeping gene. Data analysis and processing were performed using Image Lab 4.1 and Fiji software.

### 2.11 Data analysis and statistics

The data are shown as averages of at least three independent experiments with error bars representing 1 standard deviation (SD). Statistical analysis was assessed using the single tailed *t* test.

## 3 RESULTS

**FIGURE 1:**
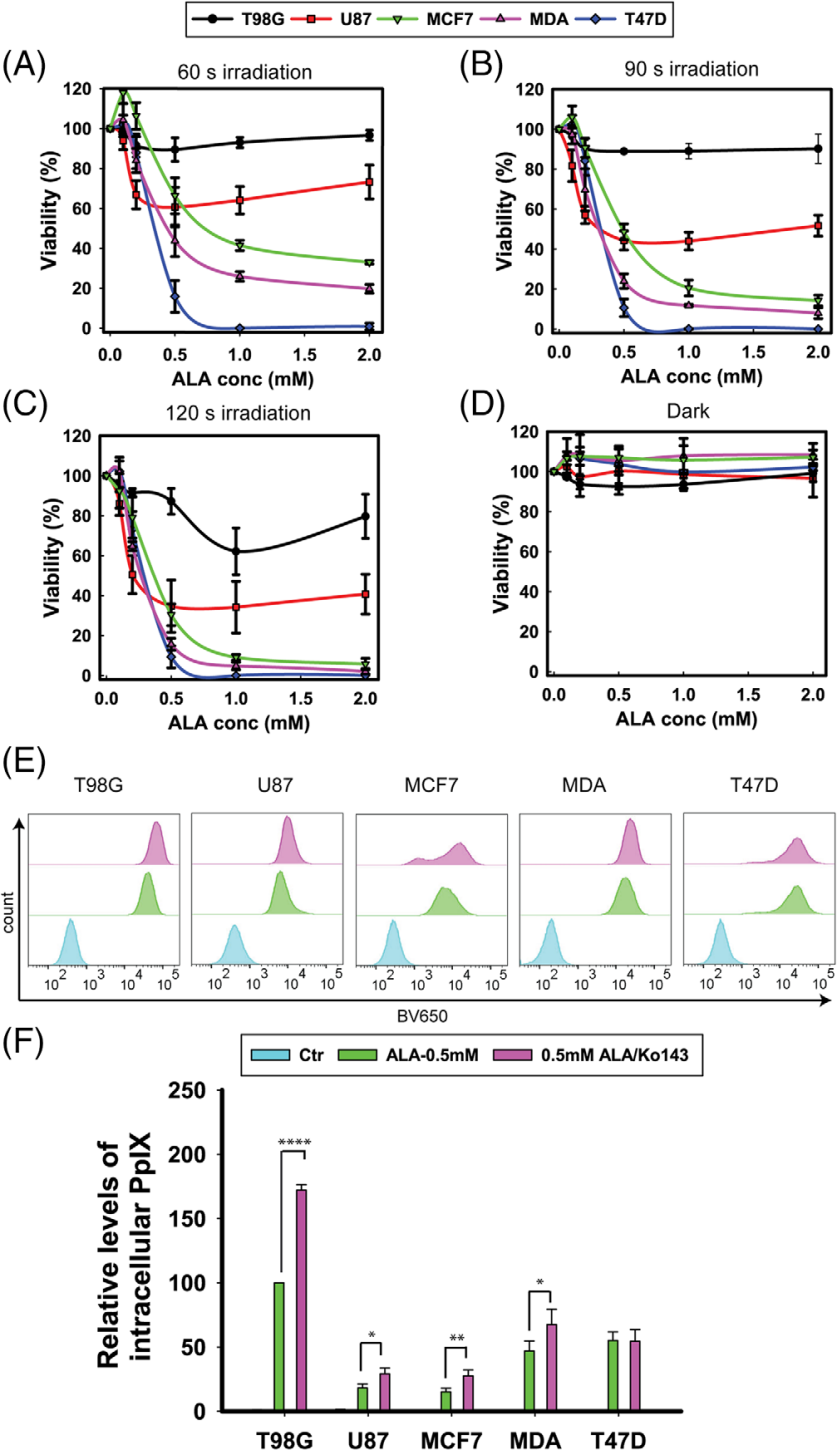
Viability of T98G, U87, MCF7, MDA‐MB‐231 (MDA), and T47D cells following 5‐ALA‐PDT vs 5‐ALA concentration and time of irradiation. Cells were incubated with 0–2 mM 5‐ALA for 4 hours, washed twice in PBS and irradiated with blue light at different time points: A, 60 seconds; B, 90 seconds; C, 120 seconds; and D, 0 s. Cell survival was measured by the MTT assay 20 hours after irradiation. The data represent the average of three individual experiments, while the error bars correspond to 1 standard deviation (SD). E, Representative histograms of 5‐ALA‐derived PpIX accumulation in T98G, U87, MCF7, MDA‐MB‐231, and T47D cells. F, Averaged quantitative data for 5‐ALA‐derived, intracellular PpIX accumulation from three independent experiments. The cells were incubated with 0.5 mM 5‐ALA for 4 hours, while the ABCG2 inhibitor Ko143 (1 μM) was introduced to the corresponding treatment groups 1 hour prior to the addition of 5‐ALA. Fluorescence was registered by flow cytometry at the BV650 channel (635 nm cut‐on longpass filter and 660/20 nm bandpass filter) following excitation at 405 nm. Statistical significance was assessed using the single tailed *t* test: (ns) *P* > .05; (*) *P* < .05; (**) *P* < .01; (***) *P* < .001; (****) *P* < .0001

The breast cancer cells were more‐responsive to 5‐ALA PDT than the GBM cells. We initially studied 5‐ALA‐PDT efficacy on the five selected cell lines through cell viability following treatment. The cell lines demonstrated diverse survival trends, which were found to be 5‐ALA concentration‐ and light dose‐dependent (Figure 1A‐C).

As shown in Figure 1A‐C, T98G (LD_30_ at 2 minutes irradiation and 1 mM 5‐ALA) and U87 (LD_30_ at 1 minute irradiation and 2 mM 5‐ALA) were the least responsive to 5‐ALA‐PDT. The MCF7 cells were the least responsive of the breast cancer cell lines (LD_30_ at 1 minute irradiation and 0.5 mM 5‐ALA), followed by MDA‐MB‐231 cells (LD_30_ at 1.5 minutes and 0.2 mM 5‐ALA). The T47D cells were the most susceptible to 5‐ALA‐PDT, as all the cells were killed at 60 seconds of irradiation, following incubation with 1 mM 5‐ALA. The collective results from the PDT experiments (Figure 1A‐C), confirm that 5‐ALA‐PDT was profoundly more photocytotoxic in the breast adenocarcinoma cell lines than in the GBM cell lines, with an order of cytotoxicity T47D > MDA‐MB‐231 > MCF7 > U87 > T98G. The dark chemical toxicity of 5‐ALA and/or the photosensitizer (PpIX) on the cells (without irradiation) is presented in Figure 1D. These data demonstrate that all the 5‐ALA concentrations studied were not toxic to the cell lines following 4 hours incubation in the dark. The data in Figure S1 portray the overlap between the emission spectrum of the lamp used for the cell irradiation and the spectral profile of the molar extinction coefficient of PpIX. The lamp's spectrum had an emission peak at around 405 nm, closely matching the PpIX absorbance Soret band (405 nm).

From these initial PDT experiments, the most suitable light exposure time across all cell lines was determined as 60 seconds, since the viability trends for the various 5‐ALA concentrations were well discriminated (Figure 1A).

### 3.1 Levels of 5‐ALA‐derived PpIX and the influence of Ko143

Subsequently, we investigated by flow cytometry the 5‐ALA‐derived PpIX production following 4 hours incubation with 0.5 mM 5‐ALA. The effect of pretreatment with Ko143, a specific inhibitor of the ABCG2 transporter on the accumulation of PpIX, was also studied. The results are presented in Figure 1E, F. Figure 1E shows the relative changes in fluorescence as histograms for each cell line and for the various treatments. The quantitative results are shown in Figure 1F and reveal considerable differences in PpIX fluorescence across the cell lines studied. The fluorescence intensity was highest in T98G (about 4 × that of U87 and MCF7, 2 × that of MDA‐MB‐31, and 1.6 × that of T47D). The addition of Ko143 increased the PpIX content in all the cell lines apart from T47D. Specifically, following incubation with 0.5 mM 5‐ALA (4 hours) the amount of PpIX was 1.8 × (*P* < .0001) higher in the presence of Ko143 for T98G, 1.7 × (*P* < .01) for U87, 1.5 × (*P* < .01) for MCF7, 1.4 × (*P* < .05) for MDA‐MB‐231, and 1 × (*P* > .05) for T47D (unaffected).

### 3.2 Effect of ABCG2 inhibitor Ko143 on 5‐ALA‐PDT

Following our findings from the PpIX production experiments, we investigated the ABCG2 transporter expression across our chosen cell line panel, incubated with 5‐ALA and before or after PDT.

**FIGURE 2:**
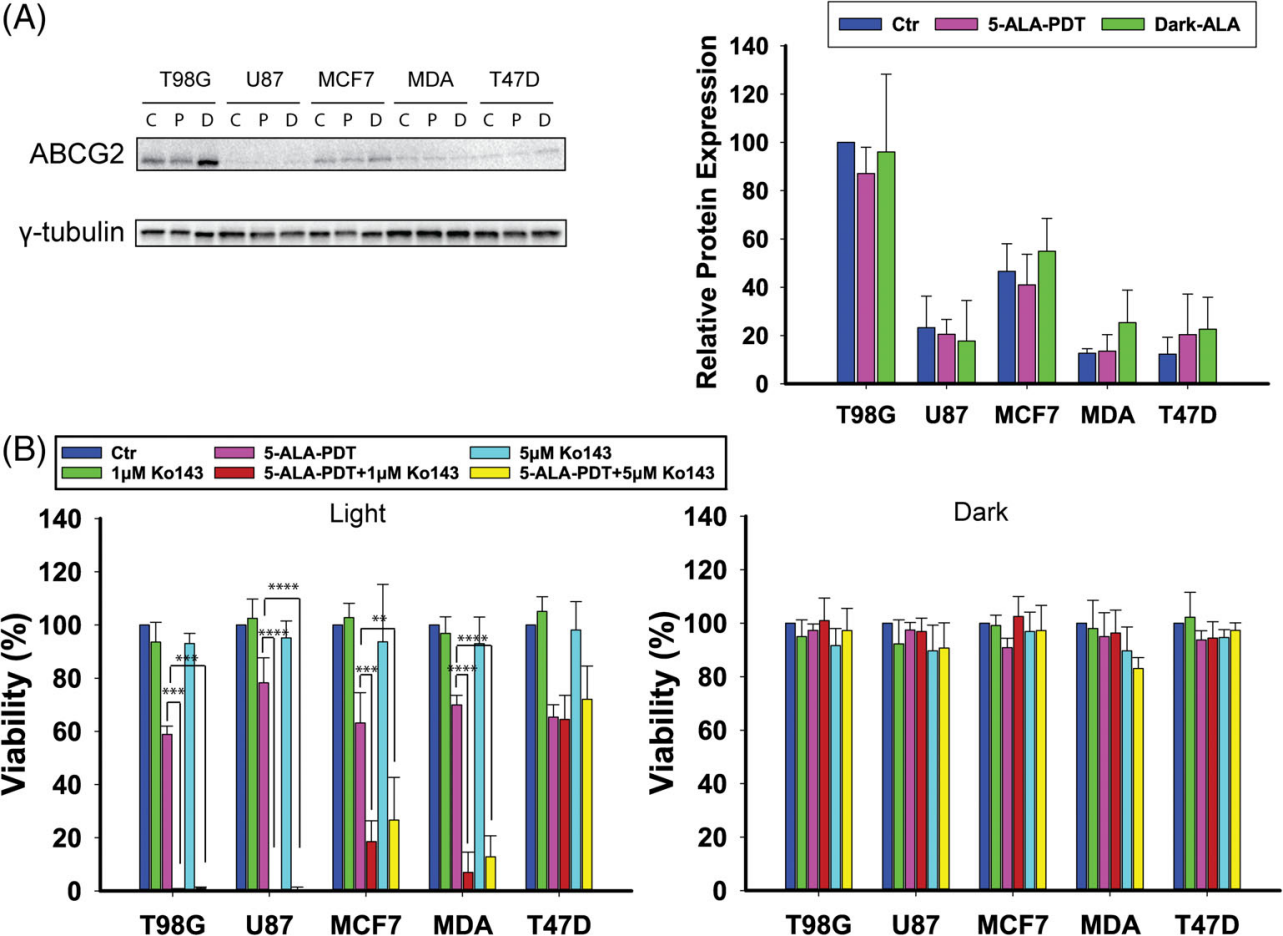
A, Western blotting analysis of ABCG2 protein levels in T98G, U87, MCF7, MDA‐MB‐231, and T47D cells after 4 hours treatment with 5‐ALA at LD_30_ concentrations for each cell line and γ‐tubulin as the loading control. There are three blotting bands per cell line: first control, second 5‐ALA‐PDT, and third 5‐ALA dark. B, Cell viability assay after 4 hours 5‐ALA treatment with or without addition of the ABCG2 inhibitor Ko143 (1 and 5 μM) 1 hour prior to 5‐ALA addition and either following PDT or in the dark. The data shown represent the average of three independent experiments, while the error bars correspond to 1 SD. Statistical significance was assessed using the single tailed *t* test: (ns) *P* > .05; (*) *P* < .05; (**) *P* < .01; (***) *P* < .001; (****) *P* < .0001

ABCG2 is an ATP‐binding cassette (ABC) transporter G2 that has been connected with regulation of the intracellular accumulation of porphyrin derivatives in cancer cells and consequently a modulator of the efficacy of PDT.^27^ Using Western blotting, we found ABCG2 expression across all the cell lines examined (Figure 2A). T98G exhibited the highest expression levels at about double that of MCF7. U87 cells expressed 4 times less ABCG2 than T98G, whereas MDA‐MB‐231 and T47D cells expressed about 8 times less ABCG2 than T98G. The ABCG2 expression levels did not change significantly following incubation with 5‐ALA in the dark or LD_30_ 5‐ALA‐PDT treatment in any of the cell lines. Following these findings, we studied the effect of the specific ABCG2 inhibitor Ko143 (1 and 5 μM, 1 hour before 5‐ALA) on 5‐ALA‐PDT across the cell lines (Figure 2B). We observed a synergistic killing effect between 5‐ALA‐PDT and Ko143 pre‐conditioning in T98G (p[1 μM] < 0.001, p[5 μM] < 0.001), U87 (p[1 μM] < 0.0001, p[5 μM] < 0.0001), MCF7 (p[1 μM] < 0.001, p[5 μM] = 0.001), and MDA‐MB‐231 (p[1 μM] = 0.0001, p[5 μM] < 0.0001) cells. On the contrary, the application of 5‐ALA‐PDT under Ko143 inhibition yielded no differential cytotoxicity in T47D cells. Incubation with Ko143 alone (1 or 5 μM) was not toxic to any of the cell lines examined.

### 3.3 Effect of ABCG2 inhibitor Ko143 on PpIX intracellular accumulation and localization

**FIGURE 3:**
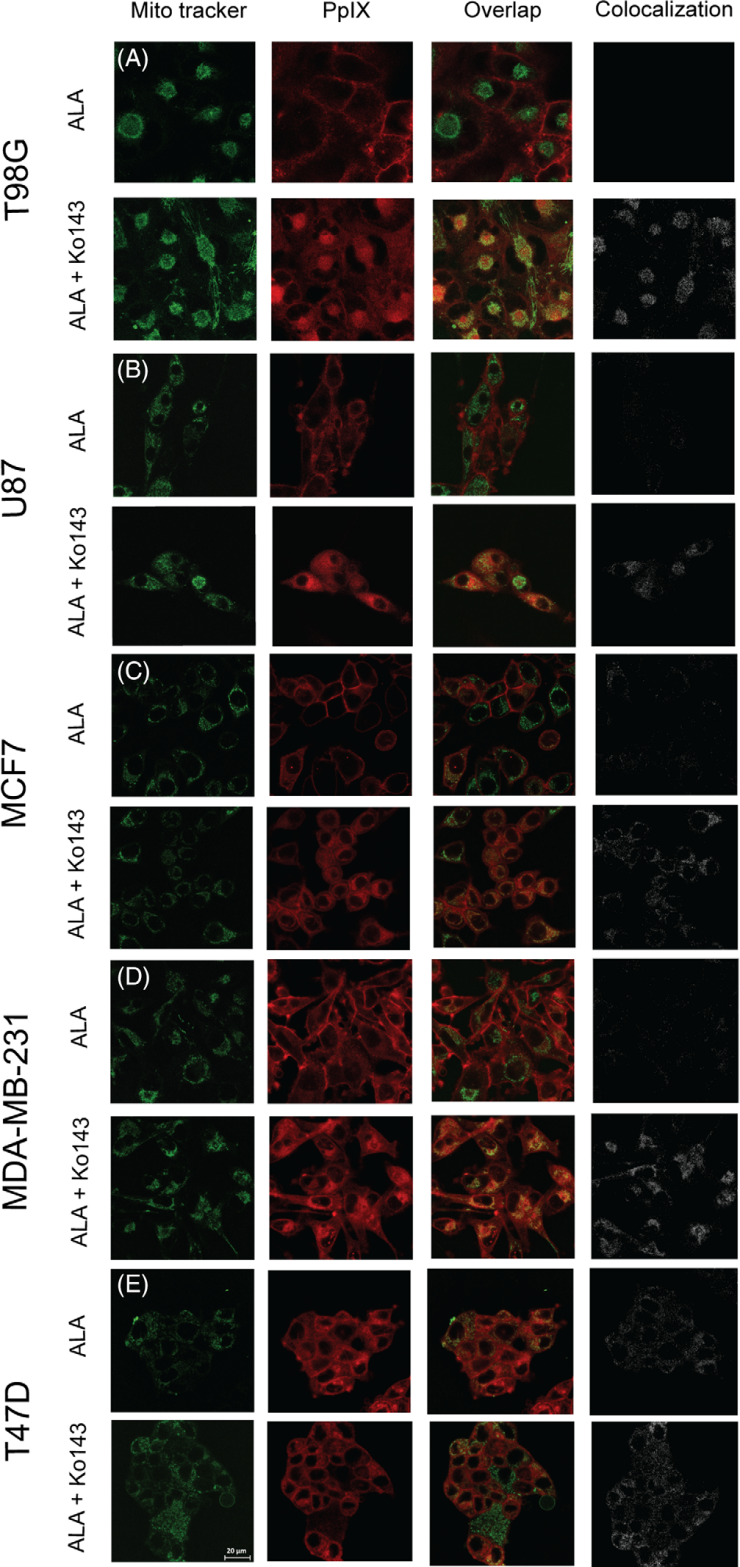
Intracellular localization of the 5‐ALA‐derived photosensitizer PpIX for the various cell lines: A, T98G; B, U87; C, MCF7; D, MDA‐MB‐231; and E, T47D. The cells were incubated with 1.5 mM 5‐ALA for 4 hours with (odd rows) or without (even rows) pretreatment with Ko143 (1 μM). The cells were incubated with 100 nm MitoTracker Green 15 minutes prior to imaging. PpIX fluorescence is represented in red (left column) and MitoTracker fluorescence in green (second column). An overlay of PpIX fluorescence with MitoTracker fluorescence is shown in the overlap panel (third column) where colocalization can be seen in yellow. Pure colocalization between PpIX and MitoTracker is shown in the fourth panel in white

To further complement these promising results, we investigated the subcellular localization of PpIX in the absence or presence of Ko143. The cells were incubated with 5‐ALA in Opti‐MEM for 4 hours, followed by the addition of MitoTracker Green FM 15 minutes before confocal imaging. As seen from the representative micrographs in Figure 3, PpIX fluorescence was observed in the cytoplasm of all the cell lines, while in some (T98G, MCF7, and MDA‐MB‐231), the fluorescence was also notable in the plasma membrane. The addition of Ko143 significantly increased the intracellular PpIX accumulation in the T98G (*P* < 0.0001), U87 (*P* < .0001), MCF7 (*P* < .0001), and MDA‐MB‐231 (*P* < .0001) cells but conferred no notable changes to PpIX fluorescence intensity in the T47D (*P* > 0.05) cells. Upon addition of Ko143, the fluorescence pattern became more punctate, primarily localizing in the perinuclear area, while the plasma membrane fluorescence component was no longer noticeable. The MitoTracker (green) fluorescence partially overlapped with the PpIX fluorescence as determined by Pearson's correlation, suggesting a slight PpIX colocalization with mitochondria in the T98G (Pearson's coefficient = 0.08, Figure 3A), U87 (Pearson's coefficient = 0.04, Figure 3B), MCF7 (Pearson's coefficient = 0.09, Figure 3C), MDA‐MB‐231 (Pearson's coefficient = 0.14, Figure 3D), and T47D (Pearson's coefficient = 0.03, Figure 3E) cells. Notably, the corresponding Pearson's coefficients were all negative in the cell groups only incubated with 5‐ALA for 4 hours, indicating no mitochondrial localization, except for the T47D cells [−0.001 for T98G, −0.007 for U87, −0.02 for MCF7, −0.02 for MDA‐MB‐231, and 0.011 for T47D].

### 3.4 Role of HO‐1 in 5‐ALA‐PDT

**FIGURE 4:**
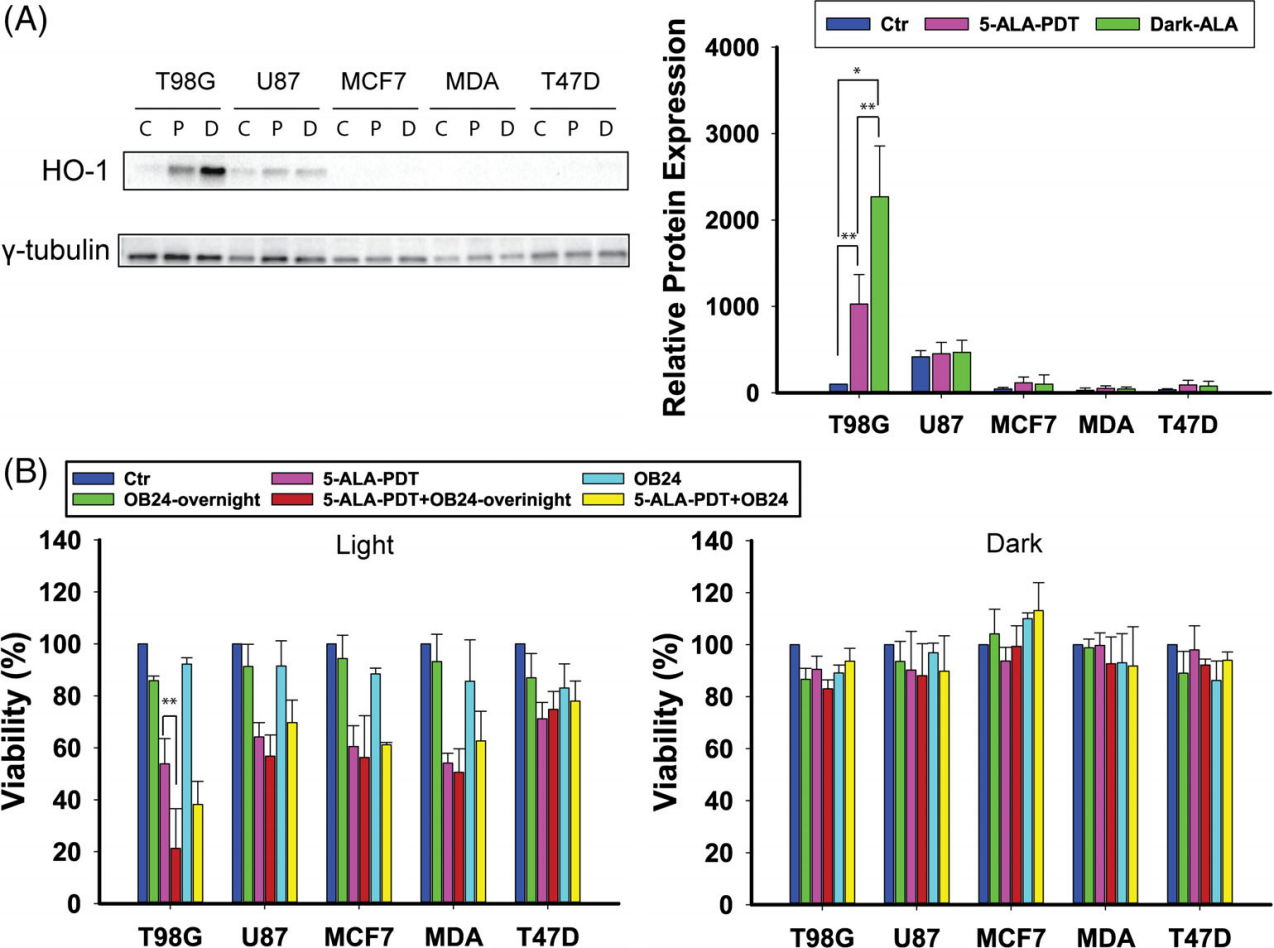
A, Western blotting analysis of HO‐1 protein levels in T98G, U87, MCF7, MDA‐MB‐231, and T47D cells after 4 hours treatment with 5‐ALA at LD_30_ concentrations for each cell line and γ‐tubulin as the loading control. There are three blotting bands per cell line: first control, second 5‐ALA‐PDT, and third 5‐ALA dark. b) Cell viability assay after 4 hours 5‐ALA treatment with or without preincubation with the HO‐1 inhibitor OB24 overnight or 1 hour prior to 5‐ALA addition, either following PDT or in the dark. The data shown represent the average of three independent experiments, while the error bars correspond to 1 SD. Statistical significance was assessed using the single tailed *t* test: (ns) *P* > .05; (*) *P* < .05; (**) *P* < .01; (***) *P* < .001; (****) *P* < .0001

HO‐1, which is induced by oxidative cellular stress,^28^ is known to catalyze heme degradation, hence mediating PDT resistance.^29^ HO‐1 expression was hardly noticeable in the non‐treated (control) T98G cells (C, 100 relative units [RU], Figure 4A), but there was a dramatic increase as a consequence of 5‐ALA administration both after light irradiation (L, × 10, *P* = .005) and under dark conditions (D, × 23, *P* = .001). HO‐1 expression in the U87 cells was nearly equal in all the groups examined (C 417|L 452|D 467 RU). HO‐1 expression was lower in the MCF7 (C 45|L 115|D 101 RU), MDA‐MB‐231 (C 28|L 51|D 43 RU), and T47D (C 35|L 90|D 77 RU) cells for the conditions under investigation.

To determine the effect of HO‐1 on 5‐ALA‐PDT, the cells were incubated with OB24, a HO‐1 inhibitor, either overnight or 1 hour prior to co‐incubation with 5‐ALA (Figure 4B). Interestingly, a synergistic effect with 5‐ALA‐PDT was only registered in the T98G cells that were preincubated overnight with 20 μM OB24 (*P* = .007) prior to 5‐ALA treatment. There was no differential cytotoxicity either in T98G for 1 hour preincubation with OB24 or in any other of the cell lines examined for both overnight and 1 hour OB24 preincubation (*P* > .05), which is in agreement with our protein expression studies and suggests that HO‐1 induction by 5‐ALA protects T98G cells against 5‐ALA‐PDT's cytotoxic effect.

### 3.5 FECH expression and the effect of its inhibition on 5‐ALA‐PDT

**FIGURE 5:**
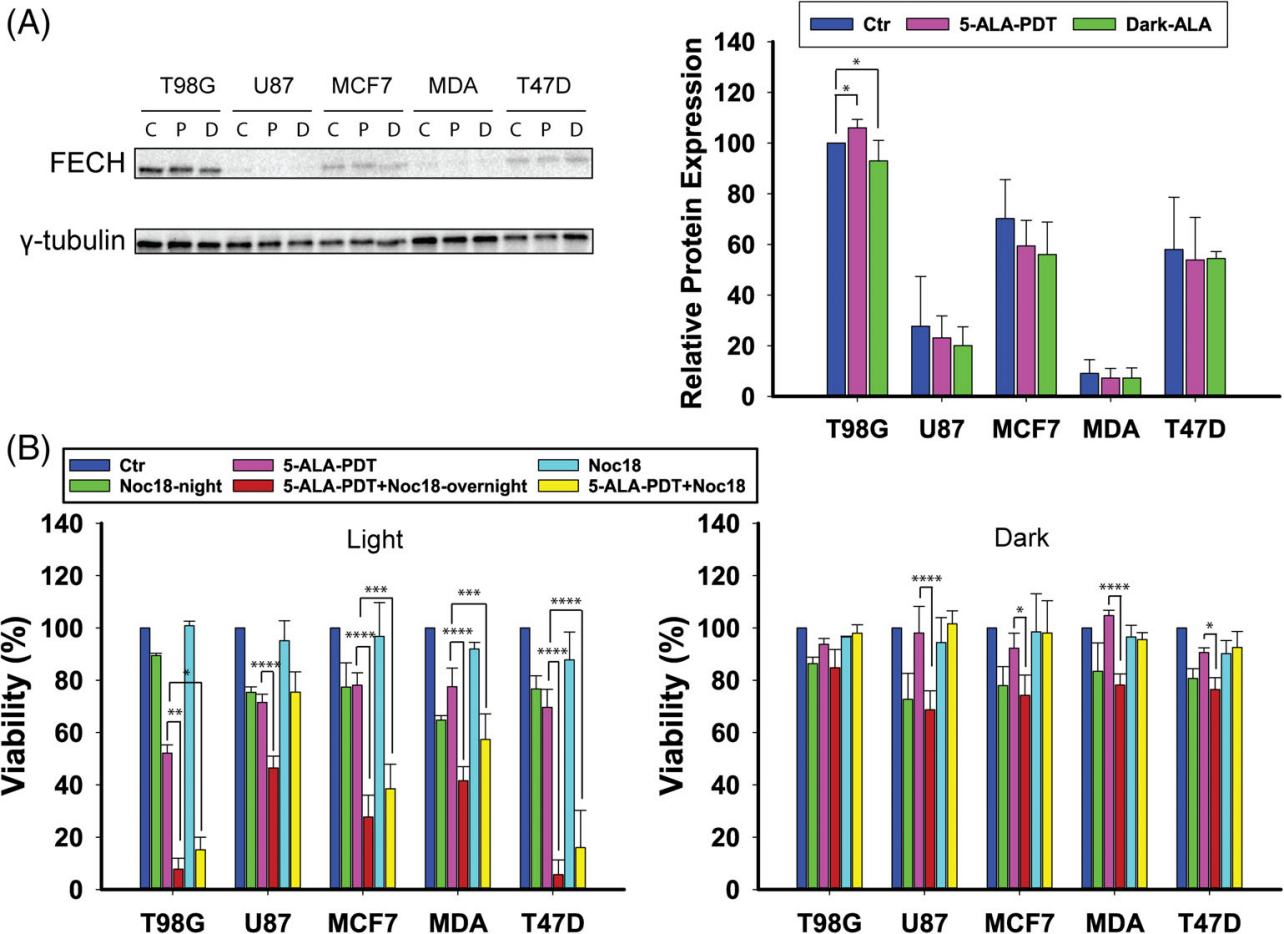
A, Western blotting analysis of FECH levels in T98G, U87, MCF7, MDA‐MB‐231, and T47D cells after 4 hours treatment with 5‐ALA at LD_30_ concentrations for each cell line and γ‐tubulin as the loading control. There are three blotting bands per cell line: first control, second 5‐ALA‐PDT, and third 5‐ALA dark. B, Cell viability after 4 hours 5‐ALA treatment with or without addition of the FECH inhibitor Noc‐18 overnight or 1 hour prior to 5‐ALA and either following PDT or in the dark. The data shown represent the average of three independent experiments, while the error bars correspond to 1 SD. Statistical significance was assessed using the single tailed *t* test: (ns) *P* > .05; (*) *P* < .05; (**) *P* < .01; (***) *P* < .001; (****) *P* < .0001

Since FECH can potentially decrease the PpIX content of the cells and consequently their responsiveness to 5‐ALA‐PDT, FECH expression was studied across the cell lines, and profound differences were found in the expression levels (Figure 5A). T98G showed the highest FECH expression (C 100|D 106|L 93 RU), MCF7 came next with C 70|L 59|D 56 RU, comparable to T47D (C 58|L 54|D 54 RU), followed by U87 (C 28|L 23|D 20 RU), and MDA‐MB‐231 (C 9|L 7|D 7 RU). There were no notable differences in the FECH expression between the control cells and the 5‐ALA treated cells either in the dark or following PDT, for any of the cell lines (*P* > .05), except between the T98G control and 5‐ALA‐PDT groups (*P* < .05), which was, however, due to the very low SDs.

To investigate the effect of FECH on 5‐ALA‐derived‐PpIX‐PDT, the cells were incubated either overnight or 1 hour prior to PDT treatment with the FECH inhibitor Noc‐18. The results are shown in Figure 5B. There was a synergistic effect between 5‐ALA‐PDT and co‐incubation with Noc‐18 in the T98G (overnight *P* < .01, 1 hour *P* = .01), MCF7 (overnight *P* < .0001, 1 hour *P* < .001) and T47D (overnight *P* < .0001, 1 hour *P* < .0001) cells, while the effects on the U87 cells and MDA‐MB‐231 were merely additive. Incubation with Noc‐18 alone was subtoxic when administered 1 hour prior to 5‐ALA treatment (0‐12%), but moderate cytotoxicity (11‐35%) was observed when cells were incubated overnight with Noc‐18.

The effects of enzymatic inhibitors Ko143, Noc 18, OB24 and BSO on the 5‐ALA‐PDT outcomes has been summarized for all cell lines in Table 1.

**TABLE 1:** Enhancement of 5‐ALA‐PDT cytotoxicity across the cell lines by the inhibitors used in the study

Cell lines	Protein/Inhibitor			
	Enhancement of 5‐ALA‐PDT cytotoxicity (%)			
	ABCG2/Ko143	HO‐1/OB24	FECH/Noc‐18	γGCS/BSO
T98G	58 ± 3	33 ± 18	44 ± 5	52 ± 14
U87	78 ± 9	7 ± 10	25 ± 6	15 ± 7
MCF7	45 ± 14	4 ± 18	50 ± 10	9 ± 8
MDA	63 ± 8	4 ± 10	36 ± 9	65 ± 11
T47D	1 ± 10	‐4 ± 9	64 ± 9	16 ± 14

### 3.6 Role of intracellular anti‐oxidants on 5‐ALA‐PDT

**FIGURE 6:**
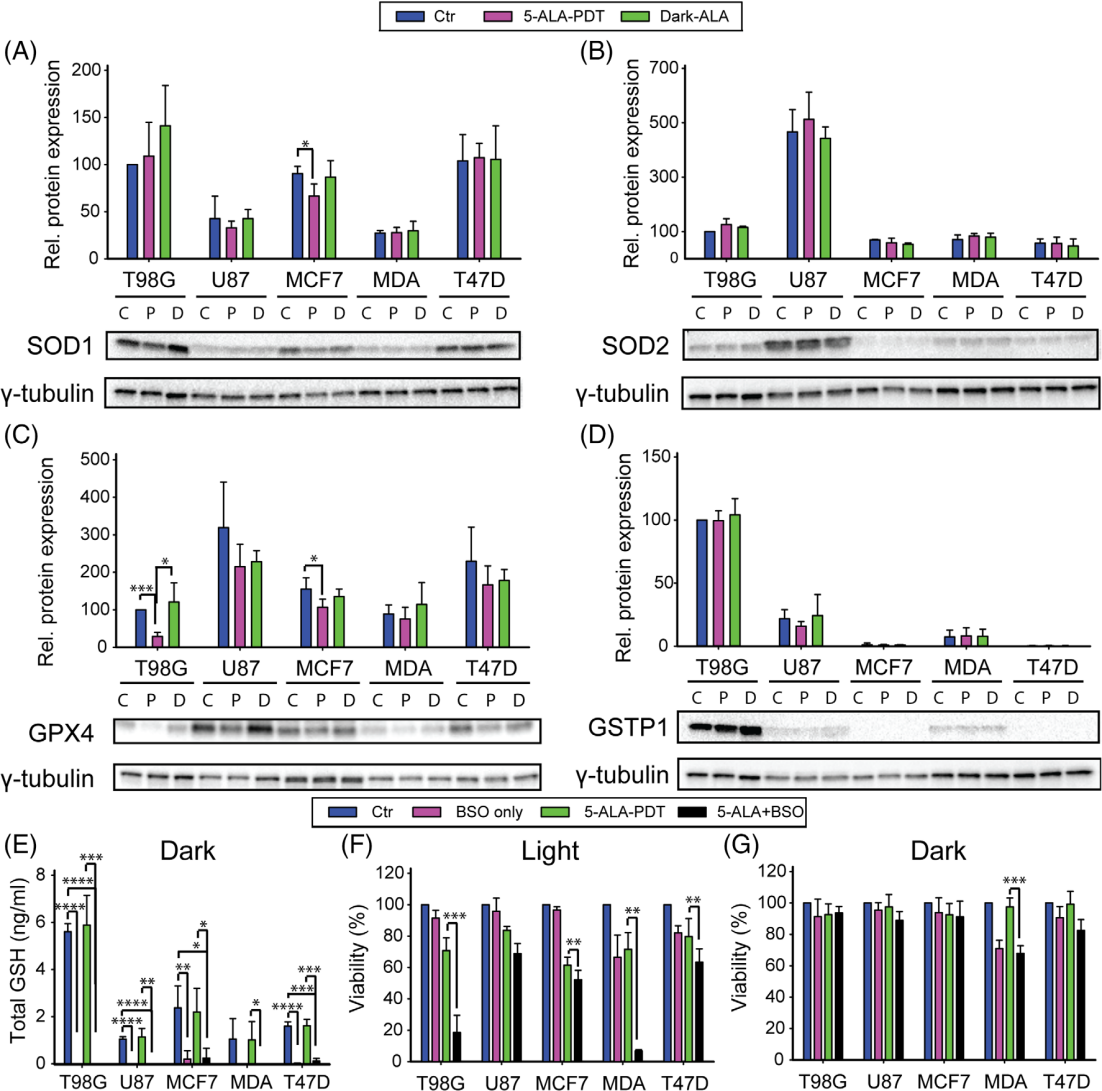
Western blotting analysis of protein levels in T98G, U87, MCF7, MDA‐MB‐231, and T47D cells after 4 hours treatment with 5‐ALA at LD30 concentrations for each cell line. γ‐tubulin was used as the loading control. The proteins examined were A, SOD1; B, SOD2; C, GSTP1; and D, GPX4. There are three blotting bands per cell line: first control, second 5‐ALA‐PDT, and third 5‐ALA dark. E, Total GSH measurement in T98G, U87, MCF7, MDA‐MB‐231, and T47D cells treated overnight with 100 μM BSO. F, Cell viability assay following 4 hours 5‐ALA treatment with or without overnight preincubation with 100 μM BSO, either following 5‐ALA‐PDT or G, in the dark. The data shown represent the average of three independent experiments, while the error bars correspond to 1 SD. Statistical significance was assessed using the single tailed *t* test: (ns) *P* > .05; (*) *P* < .05; (**) *P* < .01; (***) *P* < .001; (****) *P* < .0001

The enzymes from the superoxide dismutase (SOD) family are essential to detoxify the ROS generated by the cells^30^ by catalyzing the dismutation of superoxide radicals into H_2_O_2_. We were interested to see the influence of the level of expression of superoxide dismutases (Zn, Cu SOD [SOD1] and Mn SOD [SOD2]) on 5‐ALA‐PDT and vice versa in the cell lines examined. Figure 6A shows that the T98G cells exhibited the highest expression of cytosolic SOD1 (C 100|L 109|D 141 RU), comparable to T47D (C 104|L 107|D 106 RU) and then MCF7 (C 90|L 67|D 87 RU), while the U87 (C 43|L 33|D 43) and MDA‐MB‐231 (C 27|L 28|D 30 RU) cells had the lowest expression.

With respect to SOD2 (mitochondrial MnSOD, Figure 6B), the U87 cells had the highest expression of SOD2 (C 467|L 513|D 443 RU) compared to the other cell lines, namely T98G (C 100|L 126|D 115 RU), MCF7 (C 70|L 59|D 53 RU), MDA‐MB‐231 (C 70|L 84|D 79 RU), and T47D (C 58|L 56|D 47 RU) (Figure 6B). These data demonstrate that there were no statistically significant differences in SOD expression between the control, dark PpIX control, and PDT treatment groups in all cell lines.

We assessed the impact of glutathione peroxidase as an antioxidant defense mechanism against 5‐ALA‐PDT and hence a modulator of the PDT outcomes. The U87 cells had the highest expression of GPX4 (C 319|L 215|D 228 RU, Figure 6C). The T47D (C 230|L 166|D 178 RU) and MCF7 (C 157|L 107|D 135 RU) cells followed in the level of expression, while the T98G (C 100|L 29|D 121 RU) and MDA‐MB‐231(C 88|L 75|D 114 RU) cells exhibited the lowest GPX4 expression. These results further suggest that the decrease in GPX4 expression between the 5‐ALA‐dark and 5‐ALA‐PDT groups was only significant in the T98G cells (*P* < .05). All the other differences in expression across the treatment groups were statistically non‐significant across all the cell lines.

In relation to the level of expression of GSTP1 across the cell lines (Figure 6D), there were no major differences in GSTP1 expression between the various treatment groups. The T98G cells exhibited the highest levels of GSTP1 expression (C 100|L 99|D 104 RU), followed by the U87 (C 22|L 16|D 24 RU) and MDA‐MB‐231 (C 7|L 8|D 8 RU) cells. The GSTP1 expression levels for the MCF7 (C 1|L 0.9|D 1 RU) and T47D (C 0.3|L 0.3|D 0.3 RU) cells were barely detectable.

### 3.7 Effect of intracellular GSH levels on 5‐ALA‐PDT

The total glutathione levels were measured in dark conditions with and without overnight BSO (an inhibitor of the *de‐novo* intracellular GSH synthesis^31^) pretreatment and with or without 5‐ALA incubation (4 hours) on all the cell lines examined. The overnight treatment with 100 μM BSO profoundly depleted intracellular GSH in all the cell lines (~100% reduction, Figure 6E), without significantly affecting cell survival in the absence of oxidative stress (Figure 6G), with the exception of MCF7, where the reduction in GSH levels was approximately 90%. Following 5‐ALA‐PDT, as shown in Figure 6F, the treatment of T98G and MDA‐MB‐231 cells with BSO had a profound synergistic effect on 5‐ALA‐PDT cytotoxicity (T98G: 70% survival → 18% survival, *P* < .001, MDA‐MB‐231:72% survival → 7% survival, *P* < .01). Correspondingly, there was a 10% difference in cell death in the T47D (*P* < .01) and MCF7 (*P* < .01) cells, while there was no statistically significant effect of GSH depletion by BSO on the outcome of 5‐ALA‐PDT in the U87 cells (*P* > .05).

### 3.8 Effect of 5‐ALA‐PDT on cell metabolism

**FIGURE 7:**
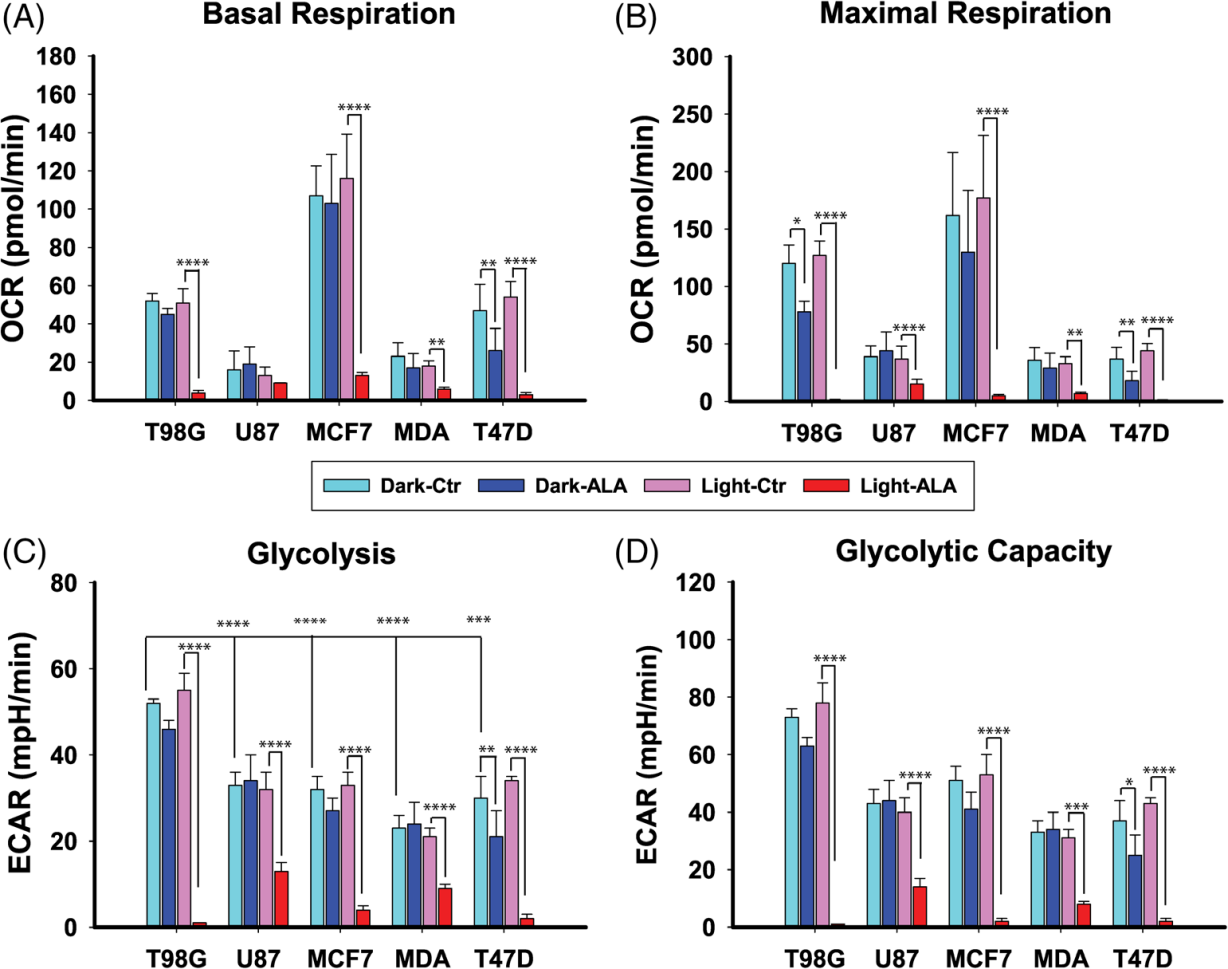
Metabolic studies 1 hour after 5‐ALA‐PDT, measured by the Seahorse XFe96 analyzer. 30 000 cells were seeded in 96 well plates. T98G, U87, MCF7, MDA‐MB‐231, and T47D cells were treated with media only or various concentrations of 5‐ALA (see methods section) for 4 hours and subsequently irradiated for 60 seconds. A, Basal oxygen consumption rate (OCR) for all cell lines and treatment groups. B, Maximal respiratory capacity as represented by the OCR following the addition of the protonophore uncoupler FCCP. C, Basal ECAR. D, Glycolytic capacity as represented by the ECAR following the addition of the F‐ATPase inhibitor oligomycin. The data shown represent the average of three independent experiments, while the error bars correspond to 1 SD. Statistical significance was assessed using the single tailed *t* test: (ns) *P* > .05; (*) *P* < .05; (**) *P* < .01; (***) *P* < .001; (****) *P* < .0001

To better understand the effect of 5‐ALA‐PDT on cell bioenergetics, we performed metabolic analysis using a Seahorse metabolic analyzer (XFe96). Under dark conditions (Figure 7A), the basal (media only) OCR of MCF7 (107 RU) was almost two times higher than that of the T98G (52 RU) and T47D (47 RU) cells, 5‐6 times higher than U87 (16 RU), and 4 times higher than the MDA‐MB‐231 (23 RU) cells. The addition of 5‐ALA to cells without irradiation only led to a substantial decrease in respiration in the T47D cells (47 → 26 RU, *P* = .009). The maximal respiratory capacity, upon addition of the protonophore FCCP, followed a similar trend, only this time apart from T47D (37 → 18 RU, *P* = .007) there was a marked difference between the dark controls and 5‐ALA dark also in T98G (120 → 78 RU, *P* = .0002) (Figure 7B). Following light irradiation in the absence of 5‐ALA, the OCR levels were in agreement with their dark counterparts. However, this was not the case for the 5‐ALA‐PDT treated cells, where OCR measurements 1 hour following irradiation demonstrated a profound drop in all the cell lines. Specifically, the basal respiratory rates (as reflected through the OCRs) exhibited reductions of 90% (*P* < .0001) for T98G, approximately 45% (*P* < .01) for U87, approximately 90% (*P* < .0001) for MCF7, approximately 75% (*P* < .001) for MDA‐MB‐231, and approximately 94% (*P* < .0001) for the T47D cells. In all cases, the cells were not responsive to the stimuli of oligo, FCCP, or MYXO 1 hour following 5‐ALA‐PDT (Supplementary Figure 3).

With regard to the glycolytic profiles of the five cell lines, the results are shown in Figure 7C. The control (media only) T98G cells were found to be the most glycolytic cell line with an extracellular acidification rate (ECAR) (52 RU) almost two times higher than the U87 (33 RU, *P* ≤ .001), MCF7 (32 RU, *P* ≤ .01), MDA‐MB‐231 (23 RU, *P* < .0001), and T47D (30 RU, *P* ≤ .001) cells. No significant drop in the extracellular acidification rate was noted after 4 hours incubation with 5‐ALA in the T98G (*P* > .05), U87 (*P* > .05), MDA‐MB‐231 (*P* > .05), and MCF7 (*P* > .05) cells. However, there was a measurable drop in the glycolytic activity of the T47D cells (30 → 21 RU, *P* < .01). After irradiation, we observed a dramatic decrease in the glycolytic rates (Figure 7D) 1 hour following 5‐ALA‐PDT: the decline was approximately 98% (*P* < .0001) for T98G, approximately 62% (*P* < .0001) for U87, approximately 88% (*P* < .0001) for MCF7, approximately 62% (*P* < .0001) for MDA‐MB‐231 and approximately 94% (*P* < .0001) for the T47D cells.

**FIGURE 8:**
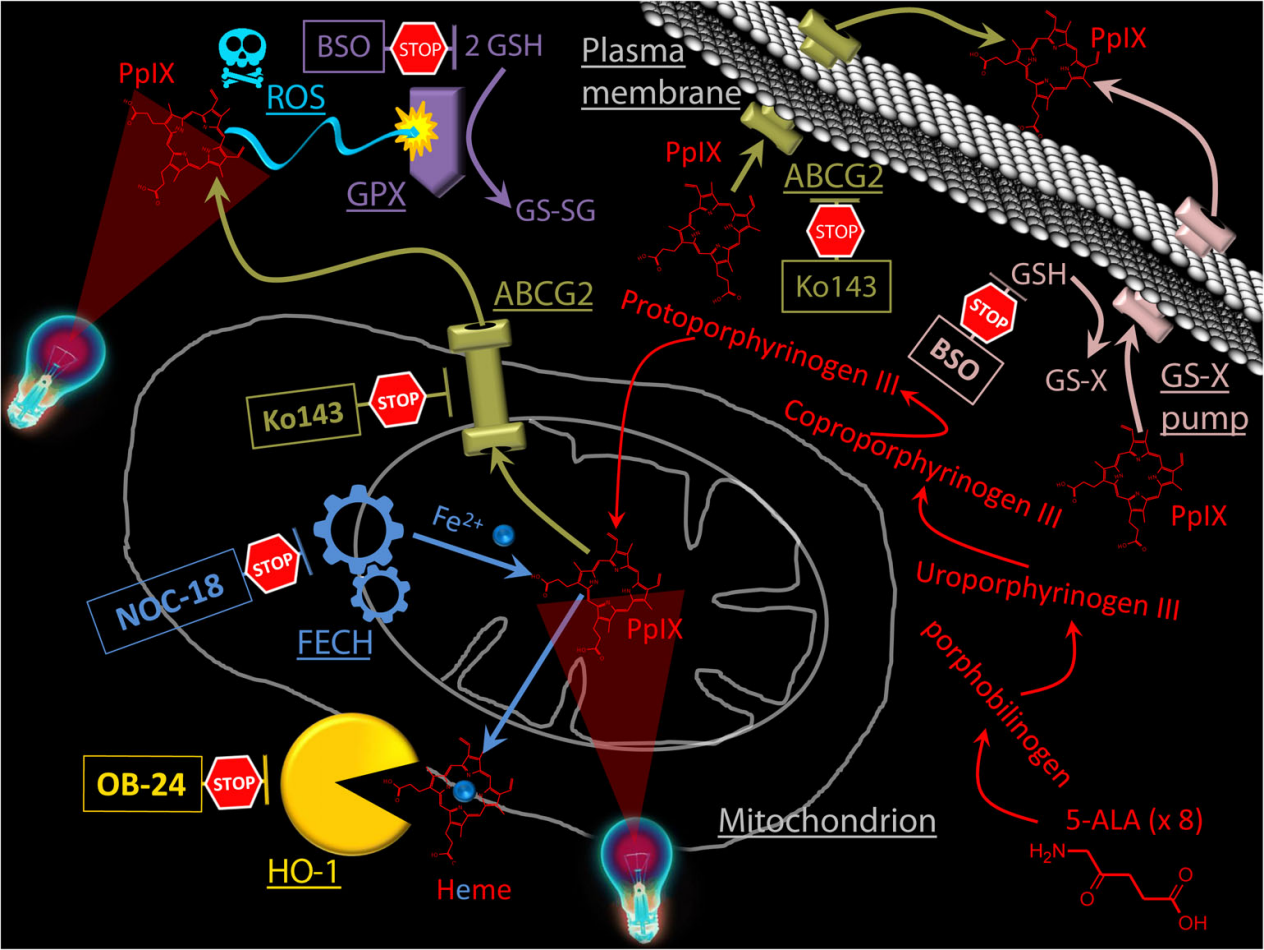
Overview of the basic biochemical processes and regulatory interventions enhancing the outcome of 5‐ALA‐PDT . The production of PpIX, the main photosensitiser in 5‐ALA‐PDT, is enhanced by the administration of exogenous 5‐ALA. The scheme above illustrates the various biochemical interventions applied in the present work aiming to enhance the 5‐ALA‐PDT outcome. BSO is shown to inhibit GSH for reasons of simplicity, whereas in reality BSO inhibits glutathione synthetase, resulting in the suppression the *de‐novo* production of GSH. GSH is a substrate for both the antioxidant activity of glutathione peroxidase (GPX) and the expulsion of xenobiotics via the GS‐X pump. Ko143 is a specific inhibitor of the ABCG2 transporters which are responsible for the expulsion of PpIX from cells and cell mitochondria. NOC 18 is an inhibitor of the enxyme ferrochelatase (FECH) which is responsible for the introduction of a Fe^2+^ into the PpIX structure transforming it into the PDT‐inept heme. OB‐24 is an inhibitor of heme oxygenase (HO‐1). HO‐1 cleaves the heme pushing the PpIX‐heme equilibrium towards the reduction of PpIX

An overview of the biochemical interactions and our proposed interventions to enhance the outcome of 5‐ALA‐PDT, as presented herein, is schematically summarized in Figure 8.

## 4 DISCUSSION

In the present study, two GBM cell lines (T98G and U87) and three breast adenocarcinoma cell lines (MCF7, MDA‐MB‐231, and T47D) exhibited different 5‐ALA‐PDT‐related cytotoxicities, with the GBM cell lines being in general more resistant (practically unresponsive). There was also a differential PDT response between the GBM cells, with T98G being more resistant than the U87 cells, which also exhibited a very limited response to 5‐ALA‐PDT. With regard to the breast cancer cell lines, MCF7 was more resistant than MDA‐MB‐231, while T47D presented as the most responsive cell line to 5‐ALA‐PDT. This confirms previous observations regarding the higher vulnerability of MDA‐MB‐231 in comparison to MCF7.^31^, ^32^ In general, the sensitivity of cell lines to PDT was found to be variant, due to phenotypic and genotypic differences as also reported elsewhere.^33^ Furthermore, there was no direct correlation between the intracellular amount of PpIX and 5‐ALA‐PDT cytotoxicity, across the cell lines (Figure S4). We further investigated these differential cytotoxicities delineated above for three reasons: (a) to understand the underlying mechanisms for these dissimilar responses; (b) to identify biomarkers for the prediction of PDT treatment outcome; and (c) to implement methods for enhancing the tumoricidal effect of 5‐ALA‐PDT based on specific tumor characteristics.

Our experimental observations warranted the investigation of possible ways to sensitize the GBM cells to 5‐ALA‐PDT, especially since it is well established that 5‐ALA‐derived PpIX fluorescence is used to precisely guide the surgical resection of GBM and bladder lesions due to its high specificity.^16^, ^17^, ^19^, ^34^, ^35^, ^36^, ^37^ Efficient GBM intraoperative 5‐ALA‐PDT could significantly prolong patients' disease‐free survival, as clinical studies have revealed that the recurrence of gliomas is frequently due to the residual cancer cells left behind after treatment.^6^ Apart from the apparent benefits for GBM cancers, the present work could also contribute by increasing the susceptibility of already responsive tumors, as illustrated through the breast cancer cell study results, auguring a more efficient treatment of a wide range of cancers in the clinical setting.

Even though the T98G cells exhibited much higher PpIX production than all the other cell lines, following administration of the same amount of 5‐ALA, they presented the lowest cytocidal response to 5‐ALA‐PDT (Figure 1A‐D). These contradictory results were later reconciled by our finding that T98G exhibited the highest expression of ABCG2 transporters. These are known to play an essential role in the regulation of the intracellular accumulation of porphyrins in cancer cells by pumping the porphyrins out of the cells and therefore reducing the efficacy of PDT.^18^ Indeed, preincubation of T98Gs with the ABCG2 inhibitor Ko143 led to a approximately 2‐fold increase in the intracellular levels of PpIX. Consequently, the T98G cells were profoundly sensitized to 5‐ALA‐PDT, following preincubation with Ko143 (Figure 2B). Apart from T98Gs, the U87, and to a lesser extent MDA‐MB‐231, cells were found to be significantly responsive to 5‐ALA‐PDT following ABCG2 inhibition by Ko143, despite the fact that these cell lines (U87 and MDA‐MB‐231) exhibited disproportionally low expression of ABCG2s. Conversely, the MCF7 cells demonstrated a moderate PDT response to ABCG2 inhibition by Ko143, despite their levels of ABCG2 expression being higher than these of U87 and MDA‐MB‐231 cells. No changes in the 5‐ALA‐PDT response was recorded for the T47D cells following ABCG2‐inhibition, even though T47Ds also expressed ABCG2 at a level comparable to MDA‐MB‐231. This can to an extent be explained by the micrographs shown in Figure 3 and Figure S2. It is evident from these representative images that different cell lines responded differently to Ko143 pretreatment. The T98G, MDA‐MB‐231, U87, and MCF7 cells exhibited enhanced mitochondrial retention of PpIX 4 hours (or even 1 hour) after Ko143 administration, while T47D showed no marked difference in the mitochondrial retention of PpIX with and without Ko143. Furthermore, the 5‐ALA‐PDT insensitivity to Ko143 also indicates that T47D recruited defense mechanisms against PDT other than ABCG2. These include FECH, as our data (Figure 5B) showed that inhibition of the latter by Noc‐18 induced a profound T47D cytotoxicity in response to 5‐ALA‐PDT. FECH is important for the production of heme since it catalyzes iron chelation by PpIX, as delineated above, but at the same time it reduces the level of PpIX available for PDT. Other mechanisms of T47D resistance to 5‐ALA‐PDT, different from those of T98G could include the high membrane‐resident GPX4 levels, which despite the relatively low levels of T47D intracellular GSH, may help catalyze the detoxification of lipid hydroperoxides, products of 5‐ALA‐PDT induced lipid peroxidation. However, even significantly reducing the GSH levels in T47Ds by overnight pretreatment with BSO does not substantially affect the PDT response, suggesting that the main defense of T47D to PDT may indeed be FECH. This lack of a multifaceted defense may also be the reason why T47D cells are most responsive to 5‐ALA‐PDT among the cell lines investigated. Other factors that are relatively enhanced in T47D cells are the antioxidant enzymes SOD1 and SOD2. By contrast, T47D seem to be relatively deficient in GSTP1 that conjugates xenobiotics with GSH exocytosis by the GS‐X pump.^38^ GSTP1 is overexpressed in a number of tumor cell lines resistant to several anticancer drugs that confer a cytoprotective role.^39^ In addition, T47Ds seem quite deficient in heme oxygenase 1 (HO‐1), a member of the heat shock protein family that has been shown to alleviate oxidative stress in tumor cells.^28^, ^40^ HO‐1 can cleave heme, thus releasing iron, which is then available to bind even more PpIX at the expense of 5‐ALA‐PDT induced photocytotoxicity.

T98Gs, however, demonstrated an adaptive HO‐1 expression, which increased with 5‐ALA administration and decreased following 5‐ALA‐PDT. Indeed, upon HO‐1 inhibition by OB24, T98G was the only cell line that exhibited enhancement of 5‐ALA‐PDT induced photocytotoxicity. In contrast, U87s, which also exhibited a moderate HO‐1 expression (not dependent upon 5‐ALA administration), did not exhibit augmented PDT cytotoxicity following OB24 pretreatment. The other cell lines only expressed minimal HO‐1 levels and no notable responses to 5‐ALA‐PDT upon OB24 pre‐conditioning. T98G, much like T47D, seemed to be responsive to FECH inhibition, as did the MCF7 cells, and their corresponding increases in PDT‐induced cell death were analogous to their reciprocal FECH levels. The U87 and MDA‐MB‐231 cells demonstrated lower FECH levels and were hence found less responsive to FECH inhibition with regard to 5‐ALA‐PDT.

With respect to antioxidant defenses, T98Gs exhibited high GSTP1 and SOD‐1 levels and the highest GSH levels on cell lines. In addition, together with MDA‐MB‐231, they were the most responsive to GSH depletion by BSO with regard to the 5‐ALA‐PDT outcome. MDA‐MB‐231 cells were found in a previous study to mostly utilize GSTP1 with GSH to detoxify hypericin^31^ and mitigate its PDT action, rather than GPX4, of which they express low levels. U87 had the highest level of SOD2, MnSOD, which is the mitochondrial SOD, and also of GPX4. These two findings indicate the high importance of cell mitochondria protection for U87, despite the fact that they exhibited the lowest OCR rates and the highest ECAR/OCR (glycolysis/respiration) ratio (approximately 2), implying a predominantly Warburg‐type cellular metabolism.

In summary, for T98G cells, adjuvant inhibition of ABCG2 alone led to a profound PDT effect, and this could also be achieved by inhibition of FECH and HO‐1 as well as intracellular GSH depletion. In the case of U87 cells, only inhibition of the ABCG2 was found to provide a synergistic adjuvant effect to 5‐ALA‐PDT (see also Table 1).

Even though the breast cancer cell lines proved to be more susceptible to 5‐ALA‐PDT, in the case of MCF7 cells, inhibition of ABCG2 or FECH greatly enhanced 5‐ALA‐PDT cytotoxicity, while for MDA‐MB‐231s, ABCG2 inhibition, and intracellular GSH depletion conferred the most profound synergies to 5‐ALA‐PDT. As also delineated earlier, the only substantial synergism to ALA‐PDT in the already responsive T47D cells was afforded by FECH inhibition. Turning these observations (also summarized in Table 1) around, the levels of expression of ABCG2, FECH primarily, but also of HO‐1, can be used as strategic biomarkers for the prediction of the treatment outcome of 5‐ALA. SOD2 can also be used as an auxiliary predictive marker, since 5‐ALA‐PDT is relevant to cell mitochondria. Finally, the triad GPX4 and GSTP1 expression vs intracellular GSH levels that have been shown to be relevant markers for PDT^31^ can be also used for 5‐ALA‐PDT. However, ABCG2, FECH, and HO‐1 are the main indicators of 5‐ALA‐PDT treatment outcome, and their inhibitors can be used in the clinic to elicit far more effective 5‐ALA‐PDT treatments.

There are numerous other inhibitors of ABCG2 besides Ko143.^41^ Many have been used in the clinic in an effort to increase the bioavailability of chemotherapeutics through barriers like the blood‐brain barrier, but they also maintain a high intracellular level of these chemotherapeutics.^41^ Apart from 5‐ALA‐PDT, ABCG2 inhibition has been found to enhance PDT for various other PSs,^42^, ^43^, ^44^ while the sensitization of a panel of TNBC cells to 5‐ALA‐PDT following ABCG2 inhibition has been shown elsewhere.^25^ Correspondingly, the inhibition of FECH has been previously shown to enhance 5‐ALA‐PDT in PC3 prostate cancer,^45^ while genetically silencing FECH has been proved to synergize with 5‐ALA‐PDT on the glioma cells SNB19.^6^ Finally, the inhibition of HO‐1 in a melanoma cell model increased their responsiveness to 5‐ALA‐PDT.^28^ Apart from these sporadic and isolated reports, however, and up to the present work, a systematic study on the various factors that could specifically synergize with 5‐ALA‐PDT to make it a more potent cancer treatment and applicable to a wider range of cancer tissues, overcoming their resistance, has been lacking.

Our study on the effect of 5‐ALA‐PDT on cell metabolism shows that there was a detrimental effect on the OCR for all cell lines with the exception of U87 cells, which did not suffer a notable loss on their basal respiration but underwent a sizable decrease in their maximal respiratory capacity. This is well in line with the observation of the fortified mitochondrial defenses of U87 (see above). It is quite possible, since PpIX is produced in mitochondria, that 5‐ALA‐PDT caused damage to one of the electron transport chain components, and severely impaired the ETC complex III much like hypericin‐PDT.^32^, ^46^ In terms of glycolysis, the U87 and MDA‐MB231 cells suffered the least damage to their glycolytic machinery among the cell lines, and since they are both metabolically more “Warburg” than “Pasteur” type of cells in terms of metabolism, perhaps clinical suppression of glycolysis prior to and even post 5‐ALA‐PDT, either pharmacologically or by regulated fasting, could lead to profound enhancement of the PDT treatment outcome.

The results of our study could provide a paradigm shift for 5‐ALA‐PDT as an anticancer treatment for two main reasons: (a) we have proposed and validated specific biomarkers that can potentially predict the outcome of 5‐ALA‐PDT treatments in the clinic based on the analysis of tumor biopsies; and (b) at the same time our results can be used to set up personalized 5‐ALA‐PDT treatments with improved efficacy based on tumor profiling with respect to our proposed biomarkers.

To translate our results into clinical practice, a two‐phase plan should be adopted. Initially, biopsies derived from the tumors of patients to be treated with 5‐ALA‐PDT should be derived and analyzed with regard to their expression of ABCG2, FECH, and/or HO‐1 against well‐known negative and positive cell lines for these markers. During that phase, the results of 5‐ALA‐PDT should be reported and correlated with the lack or abundance of the suggested biomarkers. In the second phase, an example of a proposed protocol would be to exploit the genomic analysis of the biopsy and apply clinically tested and approved inhibitors (eg, febuxostat or elacridar for ABCG2) either alone or in combination, depending on how many of the predictive biomarkers come out positive prior to PDT in order to curb the main tumor resistance with respect to the treatment.

## CONFLICT OF INTEREST

The authors declare no potential conflicts of interest.

## AUTHOR CONTRIBUTIONS

**Maria Mastrangelopoulou** Data curation‐Lead, Formal analysis‐Equal, Investigation‐Lead, Validation‐Equal, Writing‐original draft‐Lead.

**Mantas Grigalavicius** Data curation‐Equal, Formal analysis‐Equal, Investigation‐Equal, Methodology‐Equal, Validation‐Equal, Writing‐review and editing‐Equal.

**Tine Henriksen‐Raabe** Data curation‐Equal, Investigation‐Equal, Validation‐Equal.

**Ellen Skarpen** Formal analysis‐Equal, Investigation‐Equal, Writing‐review and editing‐Equal.

**Petras Juzenas** Investigation‐Supporting, Writing‐review and editing‐Supporting.

**Qian Peng** Investigation‐Supporting, Writing‐review and editing‐Supporting.

**Kristian Berg** Formal analysis‐Equal, Funding acquisition‐Equal, Project administration‐Supporting, Resources‐Equal, Writing‐review and editing‐Equal.

**Theodossis A. Theodossiou** Conceptualization‐Lead, Formal analysis‐Lead, Funding acquisition‐Lead, Methodology‐Lead, Project administration‐Lead, Resources‐Lead, Supervision‐Lead, Investigation‐Supporting, Validation‐Equal, Visualization‐Lead, Writing‐original draft‐Lead, Writing‐review and editing‐Equal.

## ETHICS STATEMENT

Only studies on commercially available cell lines were performed in the present work and hence no ethical considerations apply.

## Supporting information


**Appendix S1.** Supporting information.

## Data Availability

The data supporting the findings of the current study will be stored in an OUS repository and be made available on reasonable request.

## References

[1] Agostinis P , Berg K , Cengel KA , et al. Photodynamic therapy of cancer: an update. CA Cancer J Clin. 2011;61:250‐281. 10.3322/caac.20114. 21617154 PMC3209659

[2] Dolmans DE , Fukumura D , Jain RK . Photodynamic therapy for cancer. Nat Rev Cancer. 2003;3:380‐387. 10.1038/nrc1071. 12724736

[3] Dougherty TJ , Gomer CJ , Henderson BW , et al. Photodynamic therapy. J Natl Cancer Inst. 1998;90:889‐905. 10.1093/jnci/90.12.889. 9637138 PMC4592754

[4] Mastrangelopoulou M , Grigalavicius M , Berg K , Menard M , Theodossiou TA . Cytotoxic and photocytotoxic effects of cercosporin on human tumor cell lines. Photochem Photobiol. 2019;95:387‐396. 10.1111/php.12997. 30107033

[5] Peng Q , Warloe T , Berg K , et al. 5‐Aminolevulinic acid‐based photodynamic therapy. Clinical research and future challenges. Cancer. 1997;79:2282‐2308. 10.1002/(sici)1097-0142(19970615)79:12<2282::aid-cncr2>3.0.co;2-o. 9191516

[6] Teng L , Nakada M , Zhao SG , et al. Silencing of ferrochelatase enhances 5‐aminolevulinic acid‐based fluorescence and photodynamic therapy efficacy. Br J Cancer. 2011;104:798‐807. 10.1038/bjc.2011.12. 21304523 PMC3048207

[7] el‐Sharabasy MM , el‐Waseef AM , Hafez MM , Salim SA . Porphyrin metabolism in some malignant diseases. Br J Cancer. 1992;65:409‐412. 10.1038/bjc.1992.83. 1558795 PMC1977583

[8] Kondo M , Hirota N , Takaoka T , Kajiwara M . Heme‐biosynthetic enzyme activities and porphyrin accumulation in normal liver and hepatoma cell lines of rat. Cell Biol Toxicol. 1993;9:95‐105. 10.1007/bf00755143. 8390914

[9] Rubino GF , Rasetti L . Porphyrin metabolism in human neoplastic tissues. Panminerva Med. 1966;8:290‐292. 5967055

[10] Schoenfeld N , Epstein O , Lahav M , Mamet R , Shaklai M , Atsmon A . The heme biosynthetic pathway in lymphocytes of patients with malignant lymphoproliferative disorders. Cancer Lett. 1988;43:43‐48. 10.1016/0304-3835(88)90211-x. 3203329

[11] Van Hillegersberg R , Van den Berg JW , Kort WJ , Terpstra OT , Wilson JH . Selective accumulation of endogenously produced porphyrins in a liver metastasis model in rats. Gastroenterology. 1992;103:647‐651. 10.1016/0016-5085(92)90860-2. 1386052

[12] Rittenhouse‐Diakun K , Leengoed HVAN , Morgan J , et al. The role of transferrin receptor (CD71) in photodynamic therapy of activated and malignant lymphocytes using the heme precursor delta‐aminolevulinic acid (ALA). Photochem Photobiol. 1995;61:523‐528. 10.1111/j.1751-1097.1995.tb02356.x. 7770514

[13] Kennedy JC , Pottier RH , Pross DC . Photodynamic therapy with endogenous protoporphyrin IX: basic principles and present clinical experience. J Photochem Photobiol B Biol. 1990;6:143‐148. 10.1016/1011-1344(90)85083-9. 2121931

[14] Inoue K , Fukuhara H , Shimamoto T , et al. Comparison between intravesical and oral administration of 5‐aminolevulinic acid in the clinical benefit of photodynamic diagnosis for nonmuscle invasive bladder cancer. Cancer. 2012;118:1062‐1074. 10.1002/cncr.26378. 21773973

[15] Fukuhara H , Inoue K , Satake H , et al. Photodynamic diagnosis of positive margin during radical prostatectomy: preliminary experience with 5‐aminolevulinic acid. Int J Urol. 2011;18:585‐591. 10.1111/j.1442-2042.2011.02789.x. 21658132

[16] Stummer W , Novotny A , Stepp H , Goetz C , Bise K , Reulen HJ . Fluorescence‐guided resection of glioblastoma multiforme by using 5‐aminolevulinic acid‐induced porphyrins: a prospective study in 52 consecutive patients. J Neurosurg. 2000;93:1003‐1013. 10.3171/jns.2000.93.6.1003. 11117842

[17] Stummer W , Stocker S , Wagner S , et al. Intraoperative detection of malignant gliomas by 5‐aminolevulinic acid‐induced porphyrin fluorescence. Neurosurgery. 1998;42:518‐525; discussion 525‐516. 10.1097/00006123-199803000-00017. 9526986

[18] Ishikawa T , Kajimoto Y , Inoue Y , Ikegami Y , Kuroiwa T . Critical role of ABCG2 in ALA‐photodynamic diagnosis and therapy of human brain tumor. Adv Cancer Res. 2015;125:197‐216. 10.1016/bs.acr.2014.11.008. 25640271

[19] Tetard MC , Vermandel M , Mordon S , Lejeune JP , Reyns N . Experimental use of photodynamic therapy in high grade gliomas: a review focused on 5‐aminolevulinic acid. Photodiagnosis Photodyn Ther. 2014;11:319‐330. 10.1016/j.pdpdt.2014.04.004. 24905843

[20] Faraz S , Pannullo S , Rosenblum M , Smith A , Wernicke AG . Long‐term survival in a patient with glioblastoma on antipsychotic therapy for schizophrenia: a case report and literature review. Ther Adv Med Oncol. 2016;8:421‐428. 10.1177/1758834016659791. 27800031 PMC5066542

[21] Hefti M , von Campe G , Moschopulos M , Siegner A , Looser H , Landolt H . 5‐aminolevulinic acid induced protoporphyrin IX fluorescence in high‐grade glioma surgery: a one‐year experience at a single institutuion. Swiss Med Weekly. 2008;138:180‐185. 10.4414/smw.2008.12077 18363116

[22] Stummer W , Reulen H‐J , Meinel T , et al. Extent of resection and survival in glioblastoma multiforme: identification of and adjustment for bias. Neurosurgery. 2008;62:564‐576; discussion 564‐576. 10.1227/01.neu.0000317304.31579.17. 18425006

[23] Siegel RL , Miller KD , Jemal A . Cancer statistics, 2020. CA Cancer J Clin. 2020;70:7‐30. 10.3322/caac.21590. 31912902

[24] Aniogo EC , Plackal Adimuriyil George B , Abrahamse H . The role of photodynamic therapy on multidrug resistant breast cancer. Cancer Cell Int. 2019;19:91. 10.1186/s12935-019-0815-0. 31007609 PMC6458738

[25] Palasuberniam P , Yang X , Kraus D , Jones P , Myers KA , Chen B . ABCG2 transporter inhibitor restores the sensitivity of triple negative breast cancer cells to aminolevulinic acid‐mediated photodynamic therapy. Sci Rep. 2015;5:13298. 10.1038/srep13298. 26282350 PMC4539603

[26] Tietze F . Enzymic method for quantitative determination of nanogram amounts of total and oxidized glutathione: applications to mammalian blood and other tissues. Anal Biochem. 1969;27:502‐522. 10.1016/0003-2697(69)90064-5. 4388022

[27] Kobuchi H , Moriya K , Ogino T , et al. Mitochondrial localization of ABC transporter ABCG2 and its function in 5‐aminolevulinic acid‐mediated protoporphyrin IX accumulation. PLoS One. 2012;7:e50082. 10.1371/journal.pone.0050082. 23189181 PMC3506543

[28] Frank J , Lornejad‐Schäfer MR , Schöffl H , Flaccus A , Lambert C , Biesalski HK . Inhibition of heme oxygenase‐1 increases responsiveness of melanoma cells to ALA‐based photodynamic therapy. Int J Oncol. 2007;31:1539‐1545. 17982681

[29] Nowis D , Legat M , Grzela T , et al. Heme oxygenase‐1 protects tumor cells against photodynamic therapy‐mediated cytotoxicity. Oncogene. 2006;25:3365‐3374. 10.1038/sj.onc.1209378. 16462769 PMC1538962

[30] Fridovich I . Superoxide dismutases: anti‐versus pro‐oxidants? Anticancer Agents Med Chem. 2011;11:175‐177. 10.2174/187152011795255966. 21182471

[31] Theodossiou TA , Olsen CE , Jonsson M , Kubin A , Hothersall JS , Berg K . The diverse roles of glutathione‐associated cell resistance against hypericin photodynamic therapy. Redox Biol. 2017;12:191‐197. 10.1016/j.redox.2017.02.018. 28254657 PMC5333531

[32] Theodossiou TA , Ali M , Grigalavicius M , et al. Simultaneous defeat of MCF7 and MDA‐MB‐231 resistances by a hypericin PDT‐tamoxifen hybrid therapy. NPJ Breast Cancer. 2019;5:13. 10.1038/s41523-019-0108-8. 30993194 PMC6458138

[33] Olsen CE , Sellevold S , Theodossiou T , Patzke S , Berg K . Impact of genotypic and phenotypic differences in sarcoma models on the outcome of photochemical internalization (PCI) of bleomycin. Photodiagnosis Photodyn Ther. 2017;20:35‐47. 10.1016/j.pdpdt.2017.08.010. 28838761

[34] Coburger J , Engelke J , Scheuerle A , et al. Tumor detection with 5‐aminolevulinic acid fluorescence and Gd‐DTPA‐enhanced intraoperative MRI at the border of contrast‐enhancing lesions: a prospective study based on histopathological assessment. Neurosurg Focus. 2014;36:E3. 10.3171/2013.11.focus13463. 24484256

[35] Jichlinski P , Leisinger HJ . Photodynamic therapy in superficial bladder cancer: past, present and future. Urol Res. 2001;29:396‐405. 10.1007/s002400100215. 11828993

[36] Samkoe KS , Gibbs‐Strauss SL , Yang HH , et al. Protoporphyrin IX fluorescence contrast in invasive glioblastomas is linearly correlated with Gd enhanced magnetic resonance image contrast but has higher diagnostic accuracy. J Biomed Opt. 2011;16:096008. 10.1117/1.3622754. 21950922 PMC3188641

[37] Stummer W , Stepp H , Wiestler OD , Pichlmeier U . Randomized, prospective double‐blinded study comparing 3 different doses of 5‐Aminolevulinic acid for fluorescence‐guided resections of malignant gliomas. Neurosurgery. 2017;81:230‐239. 10.1093/neuros/nyx074. 28379547 PMC5808499

[38] Ishikawa T . The ATP‐dependent glutathione S‐conjugate export pump. Trends Biochem Sci. 1992;17:463‐468. 10.1016/0968-0004(92)90489-v. 1455517

[39] Laborde E . Glutathione transferases as mediators of signaling pathways involved in cell proliferation and cell death. Cell Death Differ. 2010;17:1373‐1380. 10.1038/cdd.2010.80. 20596078

[40] Alaoui‐Jamali MA , Bismar TA , Gupta A , et al. A novel experimental heme oxygenase‐1‐targeted therapy for hormone‐refractory prostate cancer. Cancer Res. 2009;69:8017‐8024. 10.1158/0008-5472.can-09-0419. 19808972

[41] Robey RW , Ierano C , Zhan Z , Bates SE . The challenge of exploiting ABCG2 in the clinic. Curr Pharm Biotechnol. 2011;12:595‐608. 10.2174/138920111795163913. 21118093 PMC3091815

[42] Abdel Gaber SA , Müller P , Zimmermann W , et al. ABCG2‐mediated suppression of chlorin e6 accumulation and photodynamic therapy efficiency in glioblastoma cell lines can be reversed by KO143. J Photochem Photobiol B, Biol. 2018;178:182‐191. 10.1016/j.jphotobiol.2017.10.035. 29156346

[43] Kim JH , Park JM , Roh YJ , Kim IW , Hasan T , Choi MG . Enhanced efficacy of photodynamic therapy by inhibiting ABCG2 in colon cancers. BMC Cancer. 2015;15:504. 10.1186/s12885-015-1514-4. 26149077 PMC4494642

[44] Robey RW , Steadman K , Polgar O , Bates SE . ABCG2‐mediated transport of photosensitizers: potential impact on photodynamic therapy. Cancer Biol Ther. 2005;4:187‐194. 15684613

[45] Fukuhara H , Inoue K , Kurabayashi A , et al. The inhibition of ferrochelatase enhances 5‐aminolevulinic acid‐based photodynamic action for prostate cancer. Photodiagnosis Photodyn Ther. 2013;10:399‐409. 10.1016/j.pdpdt.2013.03.003. 24284092

[46] Theodossiou TA , Papakyriakou A , Hothersall JS . Molecular modeling and experimental evidence for hypericin as a substrate for mitochondrial complex III; mitochondrial photodamage as demonstrated using specific inhibitors. Free Radic Biol Med. 2008;45:1581‐1590. 10.1016/j.freeradbiomed.2008.09.015. 18852042

