# Supplementary material for: Predictive biomarkers for 5‐ALA‐PDT can lead to personalized treatments and overcome tumor‐specific resistances
Source: Cancer Rep (Hoboken). 2020 Jul 31;5(12):e1278. doi: 10.1002/cnr2.1278 (PMC9780429; doi:10.1002/cnr2.1278)
Supplement: Supplementary file 1 — Appendix S1. Supporting information. [file CNR2-5-e1278-s001.docx]

**“Predictive biomarkers for 5-ALA-PDT can lead to personalized treatments and overcome** **tumor-specific resistances”**

Maria Mastrangelopoulou^1^, Mantas Grigalavicius^1^, Tine Henriksen Raabe^1^, Ellen Skarpen^2^, Petras Juzenas^3^, Qian Peng^3^, Kristian Berg^1^, Theodossis A. Theodossiou^1^*

^1^Department of Radiation Biology, Institute for Cancer Research, Oslo University Hospital, Oslo, Norway.

^2^Department of Molecular Cell Biology, Institute for Cancer Research, Oslo University Hospital, Oslo, Norway.

^3^Department of Pathology, The Norwegian Radium Hospital, Oslo University Hospital, 0379 Oslo, Norway

**Supplementary information**

**Supplementary Fig. 1.** Spectral profile of PpIX and emission profile of irradiation source.

**Supplementary Fig. 2.** Intracellular localization of the 5-ALA-derived photosensitizer PpIX for the various cell lines.

**Supplementary Fig. 3.** Metabolic studies 1 h after 5-ALA-PDT, measured by the Seahorse XFe96 analyzer.

Supplementary Figure 1


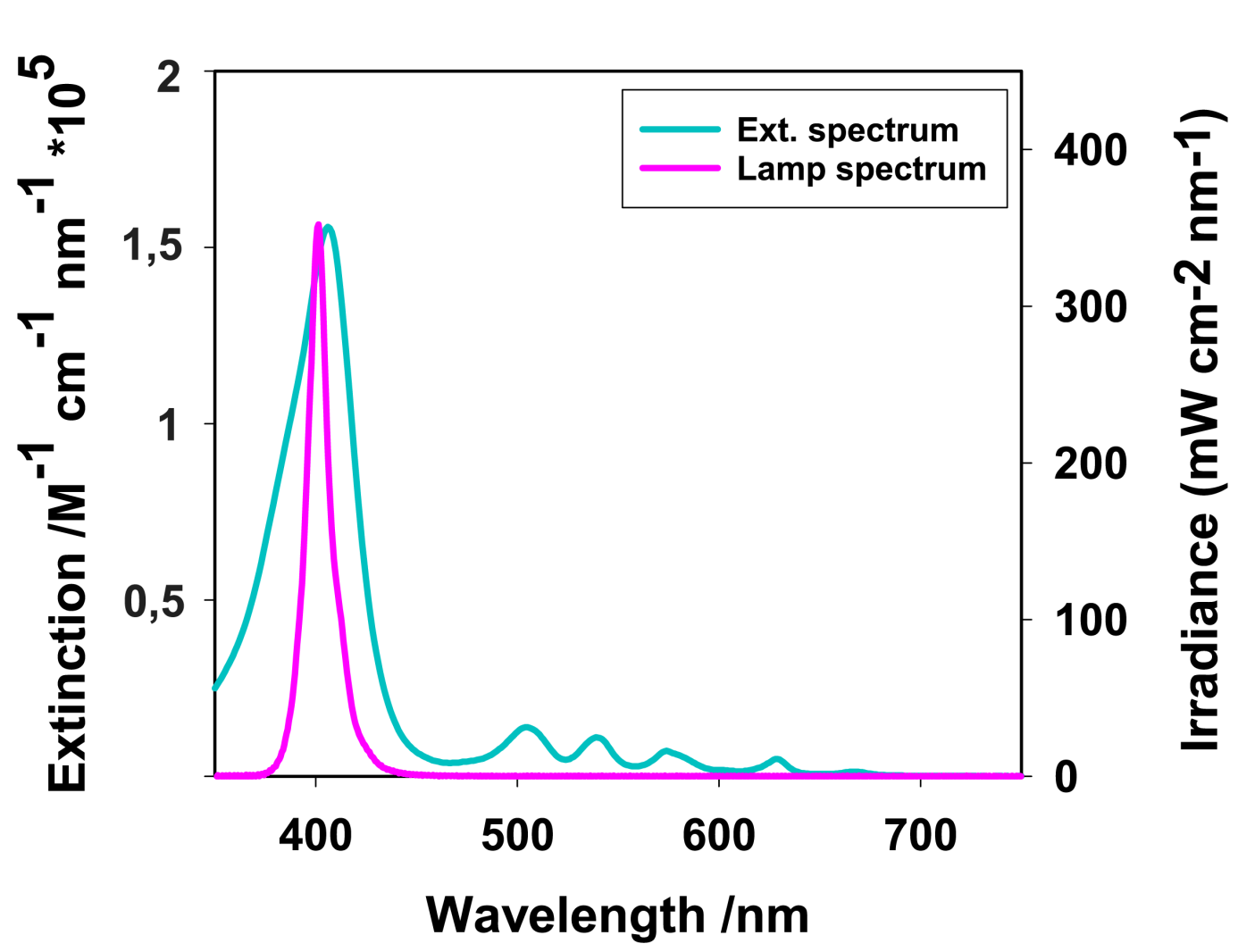


**Supplementary Fig.1.**  Spectral profile of the molar extinction coefficient of PpIX in DMSO (blue line) vs. the emission spectrum of our lamp built in-house (yellow line). The PpIX absorbance profile also exhibited four Q bands at ~510, 543, 583, and 633 nm.

| 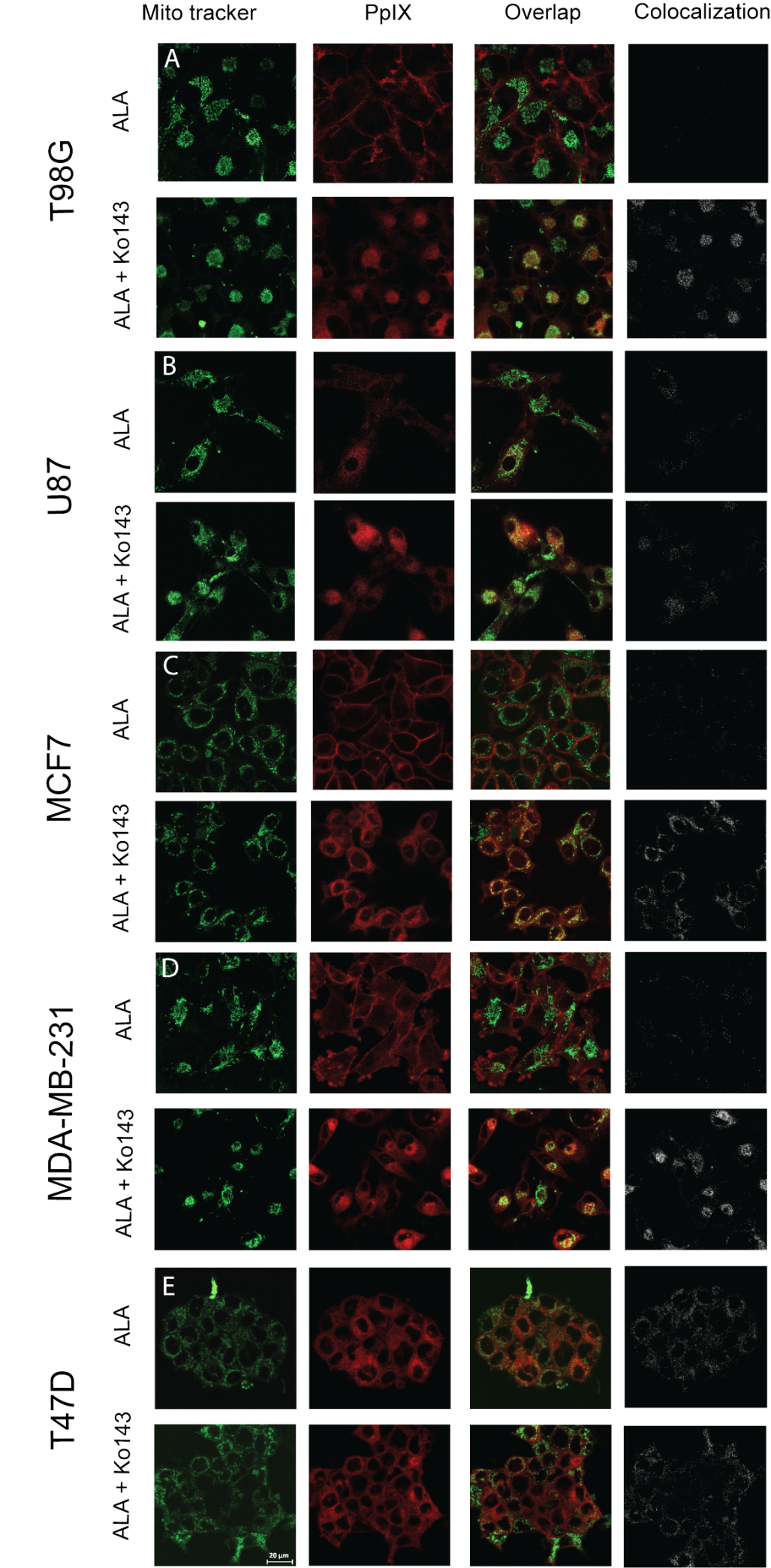 |
| --- |
|  |

**Supplementary Fig. 2.** Intracellular localization of the 5-ALA-derived photosensitizer PpIX for the various cell lines: a) T98G, b) U87, c) MCF7, d) MDA-MB-231, and e) T47D. Cells were incubated with 1.5 mM 5-ALA for 1 h with (even rows) or without (odd rows) pretreatment with Ko143 (1 µM). The cells were incubated with 100 nm MitoTracker Green 15 min prior to imaging. MitoTracker Green fluorescence is represented in green (left column) and PpIX fluorescence in red (second column). An overlay of PpIX fluorescence with MitoTracker fluorescence is shown in the overlap panel (third column) where colocalization can be seen in yellow. Pure colocalization between PpIX and MitoTracker is shown in the colocalization panel in white (fourth panel).


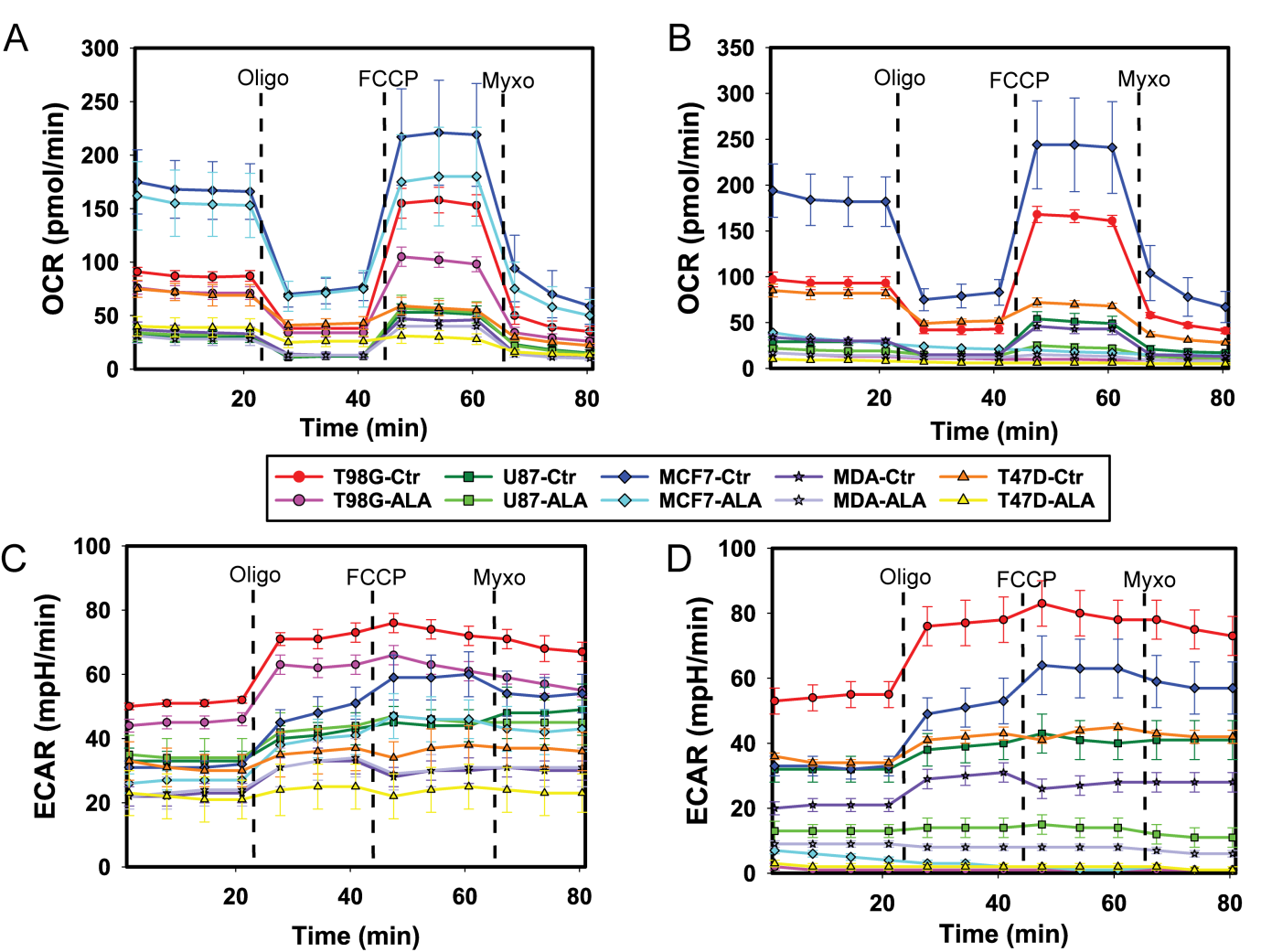


**Supplementary Fig. 3.** Metabolic studies 1 h following 5-ALA-PDT, measured by the Seahorse XFe96 analyzer. 30,000 cells (T98G, U87, MCF7, MDA-MB-231, and T47D) per 96-well plate were treated with media only or various concentrations of 5-ALA corresponding to LD_30_ for 4 h and subsequently irradiated for 60 sec. Basal oxygen consumption rates (OCRs) for all cell lines without and with light irradiation are presented in panels a and b, respectively. The basal extracellular acidification rate (ECAR) for all cell lines without and with light irradiation are presented in panels c and d, respectively. The data shown represent the average of three independent experiments, while the error bars correspond to 1 SD.

**
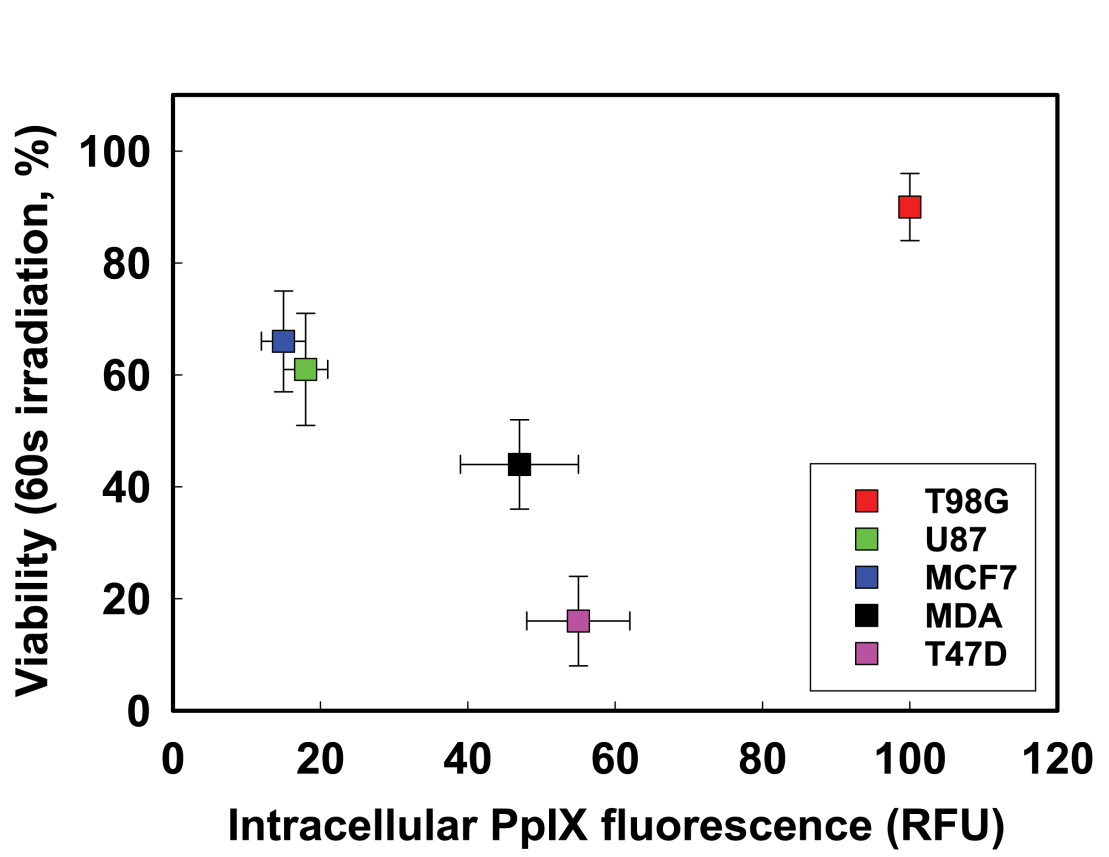
**

**Supplementary Fig. 4.** Intracellular production of PpIX f llowing 4h incubation with 0.5mM 5-ALA (taken from the flow data, Fig.1F) vs. cell viability following 60s irradiation again after incubation with 0.5mM 5-ALA (data taken from Fig. 1A). Data shown represent the average of three independent experiments, while the error bars correspond to 1 SD.
